# A PI-Dual-STGCN Fault Diagnosis Model Based on the SHAP-LLM Joint Explanation Framework

**DOI:** 10.3390/s26020723

**Published:** 2026-01-21

**Authors:** Zheng Zhao, Shuxia Ye, Liang Qi, Hao Ni, Siyu Fei, Zhe Tong

**Affiliations:** 1 School of Automation, Jiangsu University of Science and Technology, Zhenjiang 212100, China; 2 Jiangsu Shipbuilding and Ocean Engineering Design and Research Institute, Zhenjiang 212100, China

**Keywords:** fault diagnosis, explainability, LLM, SHAP, explainable AI

## Abstract

This paper proposes a PI-Dual-STGCN fault diagnosis model based on a SHAP-LLM joint explanation framework to address issues such as the lack of transparency in the diagnostic process of deep learning models and the weak interpretability of diagnostic results. PI-Dual-STGCN enhances the interpretability of graph data by introducing physical constraints and constructs a dual-graph architecture based on physical topology graphs and signal similarity graphs. The experimental results show that the dual-graph complementary architecture enhances diagnostic accuracy to 99.22%. Second, a general-purpose SHAP-LLM explanation framework is designed: Explainable AI (XAI) technology is used to analyze the decision logic of the diagnostic model and generate visual explanations, establishing a hierarchical knowledge base that includes performance metrics, explanation reliability, and fault experience. Retrieval-Augmented Generation (RAG) technology is innovatively combined to integrate model performance and Shapley Additive Explanations (SHAP) reliability assessment through the main report prompt, while the sub-report prompt enables detailed fault analysis and repair decision generation. Finally, experiments demonstrate that this approach avoids the uncertainty of directly using large models for fault diagnosis: we delegate all fault diagnosis tasks and core explainability tasks to more mature deep learning algorithms and XAI technology and only leverage the powerful textual reasoning capabilities of large models to process pre-quantified, fact-based textual information (e.g., model performance metrics, SHAP explanation results). This method enhances diagnostic transparency through XAI-generated visual and quantitative explanations of model decision logic while reducing the risk of large model hallucinations by restricting large models to reasoning over grounded, fact-based textual content rather than direct fault diagnosis, providing verifiable intelligent decision support for industrial fault diagnosis.

## 1. Introduction

The rapid development of IoT technology has continuously improved the availability of complex equipment data, creating new opportunities for exploring new methods and tools for mechanical predictive maintenance. The core objective of predictive maintenance is to predict or detect mechanical failures in a timely manner to avoid losses. For a long time, fault diagnosis has been a hot topic in the field of equipment health management, especially in the field of rotating machinery. As a core component of various types of rotating machinery, gears are widely used in key industries such as aerospace, automobile manufacturing, and maritime transportation. However, such systems often operate under harsh conditions such as high pressure, strong vibrations, and extreme temperatures, which can easily lead to mechanical fatigue, wear, and other abnormalities, triggering sudden failures or even chain shutdown accidents, resulting in significant economic losses and safety risks. Therefore, implementing effective gear fault diagnosis is crucial for ensuring the stable operation of mechanical equipment.

Meanwhile, as the pace of Industry 4.0 and 5.0 accelerates, the integration of artificial intelligence (AI) with industrial environments is becoming increasingly seamless, particularly achieving significant advancements in fault diagnosis. However, in the diagnosis of complex equipment failures, deep learning models generally suffer from issues such as opacity and weak interpretability. The “black-box” nature of these methods hinders practical implementation as trustworthiness, model interpretability, and decision interpretability are critical for informed decision-making.

Additionally, some AI regulatory frameworks, such as the EU AI Act, make it impractical to directly implement opaque AI in critical industrial tasks.

Existing gear fault diagnosis algorithms can primarily be categorized into knowledge-driven methods and data-driven methods. Knowledge-driven fault diagnosis methods focus on understanding the mechanisms behind fault occurrence, manually extracting physically meaningful features using signal processing techniques, and leveraging domain expert knowledge to construct diagnostic rule databases or fault tree models, ultimately determining the fault state [1,2]. Knowledge-driven methods have strong reasoning capabilities and good robustness and demonstrate strong interpretability in scenarios where the equipment mechanism is well understood. However, this method has the limitation of being highly dependent on human experience for knowledge acquisition, making it difficult to apply to complex, nonlinear, and non-stationary systems such as ship main propulsion systems. The demand for real-time performance and high efficiency has driven researchers to explore data-driven fault diagnosis methods. These methods employ various intelligent algorithms combined with signal processing techniques to automatically extract high-dimensional fault features from equipment and classify them, ultimately achieving fault diagnosis [3,4]. Data-driven methods do not overly rely on expert knowledge, can handle more complex machinery, and achieve higher diagnostic accuracy, making them widely applicable in fault diagnosis [5,6,7,8,9]. For instance, recent studies have combined signal processing techniques (e.g., wavelet analysis) with intelligent models to enhance fault feature extraction: Melluso et al. utilized wavelet-supported processing to extract torque fault signals in hybrid electric powertrains, demonstrating effective fault information mining for complex systems [10]; Chen et al. further proposed an interpretable wavelet-based model for spatiotemporal feature extraction in fault diagnosis, addressing the opacity issue of traditional data-driven models to some extent [11]. However, this method requires a large amount of fault sample data for feature extraction and classification, severely limiting its application in data-scarce industrial scenarios. Additionally, issues such as the inherent opacity of deep learning models and weak result interpretability severely restrict their practical application in risk-sensitive scenarios.

To address the issue of limited sample data constraining the engineering application of fault diagnosis technology, scholars typically seek solutions from two directions: utilizing external related data and mining internal related information. Some studies attempt to use external data related to the target data to assist in diagnosis. Specifically, they use a large amount of external associated data to train the model’s feature extraction capabilities then fine-tune the model using a small amount of target data to enable it to master high-dimensional feature extraction and classification capabilities. Transfer learning is a typical method for fault diagnosis using external associated data [12,13]. Another group of studies focuses on deeply mining the fault information inherent in the target data itself. For complex devices, the different signals they collect inevitably have intrinsic correlations, and this correlation information can be used for fault diagnosis. Graph neural networks (GNNs) are among the representative algorithms for diagnosing faults by mining the intrinsic correlations in data [14,15]. However, it is worth noting that existing GNN-based methods still have limitations: first, the physical characterization ability of constructing sensor relationship graphs based on signal similarity is insufficient; second, these models mainly focus on spatial correlation while neglecting the temporal dimension.

When addressing issues such as the lack of transparency in the diagnostic process of deep learning models and the weak interpretability of diagnostic results, scholars often refer to the “black-box” nature of DL models, which is a long-standing problem. There have been numerous attempts to enhance the interpretability of models [16]. However, in different research papers, the concept of explainability has been ambiguous, as mentioned in the review [17], which refers to “interpretability, explainability, understandability, comprehensibility, and transparency.” Current researchers have made a clear distinction between models that are designed to be explainable and models that can be explained using external XAI techniques. This binary distinction is also seen as the difference between explainable models and model explainability techniques. Explainable models focus on the explainability attributes of the model itself, typically selecting transparent models with clear logic, such as logistic regression, decision trees, K-Nearest Neighbors, and Bayesian models, which inherently convey a certain degree of explainability [18,19]. In addition to using transparent models, some scholars also incorporate external knowledge into the construction process of deep learning models, using physical information networks to enhance the model’s inherent explainability [20]. Model explainability techniques, also known as post hoc explainability techniques, do not involve designing explainable models but rather use various methods to visualize the model’s behavior and working logic, assisting non-experts in understanding the model’s decision-making process or rationality to enhance model explainability [21]. Common methods include textual explanations [22], visual explanations [23], local explanations [24], simplified explanations [25], and feature correlation techniques [26]. Among these, the SHAP (Shapley Additive Explanations) framework proposed by Lundberg et al. in 2017 has garnered significant attention from both academia and industry due to its robust game theory foundation, broad model applicability, and ability to unify multiple explanation methods [27]. However, current academic research rarely combines the explainability of the model itself with post hoc explainability techniques to jointly enhance model explainability. Additionally, the review [28] mentions that XAI researchers often rely on their intuition to determine reasonable explanations without prior consultation with experts. When inconsistencies with intuitive understanding are detected, confusion arises, leading to doubts about how explainability is generated. It is evident that the majority of post hoc explainability technique research has only utilized explainability techniques without evaluating rationality.

Large language models (LLMs) have emerged as a key research focus in recent years, thanks to their robust reasoning capabilities, adaptability, and versatility, which have led to their widespread application across various fields of life. Some scholars have already begun exploring the use of large models in fault diagnosis research [29]. Lin et al. conducted modal alignment training on descriptive texts of engineering data and equipment operational states in the feature space, activating the LLM’s ability to understand time series data modalities, and proposed a novel fault diagnosis method based on multi-modal large language models, termed FD-LLM [30]. Ma et al. addressed the limitations of LLMs in terms of their ability to absorb new knowledge, the generation of hallucinations, and transparency by proposing a novel fault diagnosis method enhanced by knowledge graphs, termed the Fault Diagnosis Reasoning Knowledge Graph LLM (FDRKG-LLM) [31]. Wang et al. leveraged the powerful reasoning capabilities of large language models, combined with contextual information from envelope spectrum graphs and expert knowledge, to propose a multimodal DiagLLM model aimed at enhancing the generality and interpretability of bearing fault diagnosis using the capabilities of multimodal large language models, ultimately achieving accurate diagnosis of bearing faults [32]. However, existing studies primarily rely on the inherent reasoning capabilities of LLMs or multimodal LLMs to directly diagnose faults by integrating expert experience with information processing techniques. Considering that large models require massive amounts of data for fine-tuning to achieve optimal diagnostic results and that, even with sufficient data, hallucination issues persist, coupled with the inherent black-box nature of direct diagnostic methods, this study focused on constructing a novel explainability framework while effectively leveraging the textual reasoning capabilities of large models.

These challenges—including low trust in black-box diagnostic models among on-site industrial engineers, unvalidated rationality of post hoc explainability results, persistent hallucinations in LLM-generated fault analysis content, and and the fundamental incompatibility of opaque systems with high-reliability industrial requirements—directly hinder the practical deployment of AI-driven fault diagnosis in critical gear-operated industrial scenarios. To address these practical industrial needs, this study sets a clear practical purpose: to develop a trustworthy, interpretable, and regulatory-aligned gear fault diagnosis framework that can support reliable on-site decision-making. Specifically, this framework is intended to enable on-site engineering personnel to comprehend and trust the diagnostic decisions of the model, to ensure the verifiable reliability of model explanation results, and to generate credible, traceable diagnostic reports that avoid unsubstantiated content. To achieve this practical objective, this paper proposes a novel PI-Dual-STGCN fault diagnosis model based on a SHAP-LLM joint explanation framework.

The architecture includes a dual-graph collaborative spatiotemporal graph convolutional (STGCN) fault diagnosis model that incorporates physical information, which aims to enhance the model’s interpretability, and a SHAP-LLM joint enhancement explanation mechanism that achieves visual visualization of the model’s working logic and text translation for post hoc interpretability. The main contributions of this study are as follows.

1. Constructing a dual-graph structure based on the physical topological relationships and signal similarity derived from sensor distributions significantly reduces reliance on data volume and computational resources for relationship inference while addressing the limitations of pure physical connections in representing implicit dynamic associations.
2. Designing multi-scale differential layers that fuse differential features with residuals from the original input effectively distinguishes structural evolution patterns across different scales, mitigating the over-smoothing issue commonly observed in graph neural networks.
3. The SHAP framework based on Deep Explainer is used to quantify the impact of diagnostic model input features on specific prediction results, achieving a dual visual interpretation of the model at both the global and local levels. Building on this, an innovative quantitative evaluation metric system for SHAP interpreters is proposed, addressing the critical issue of the lack of objective assessment standards for interpretation results in existing methods.
4. Combining SHAP quantitative information with expert knowledge and fault diagnosis results, a private knowledge base for fault diagnosis is constructed. Using RAG retrieval enhancement technology and feature prompting engineering, high-dimensional SHAP values are mapped into structured semantic inputs to drive LLMs to generate credible text explanations aligned with domain knowledge, establishing a domain-knowledge-guided text generation mechanism for fault diagnosis explanations.

## 2. Materials and Methods

**Figure 1:**
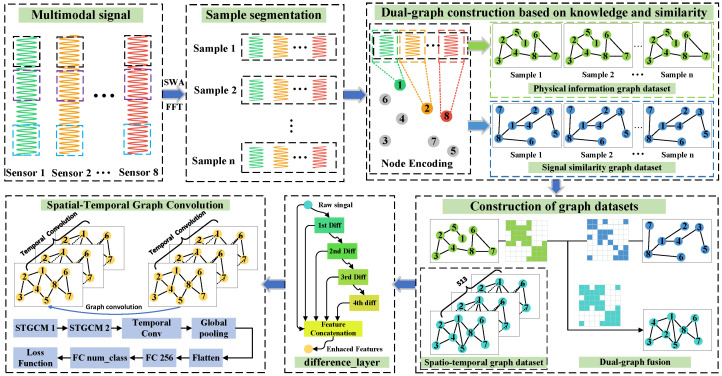
Fault diagnosis model embedded with physical information.

**Figure 2:**
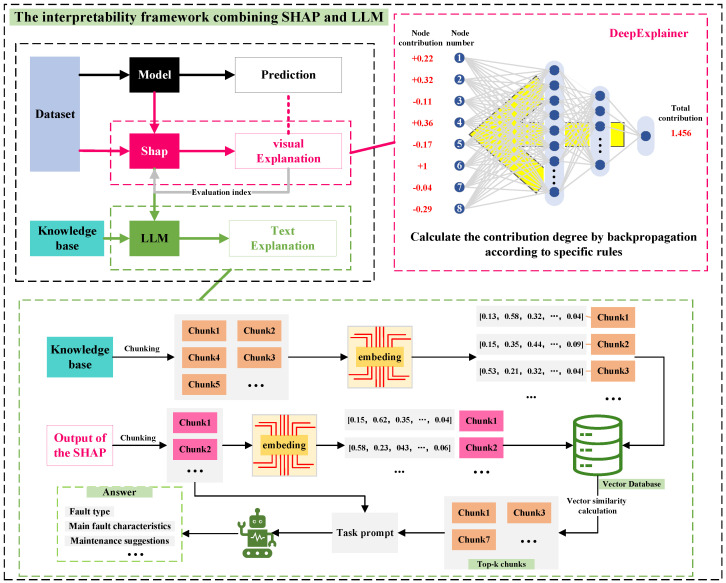
The explanation framework combining SHAP and LLM.

The PI-Dual-STGCN fault diagnosis model based on the SHAP-LLM joint explanation framework mentioned in this paper consists of two parts. The first part is the PI-Dual-STGCN fault diagnosis framework, which is used for fault diagnosis tasks. The second part is the SHAP-LLM joint explanation framework, which is used for fault diagnosis logic explanation tasks, as shown in  Figure 1 and  Figure 2, respectively.

 Figure 1 shows a spatiotemporal graph convolutional neural network driven by physical and data dual graphs. This model introduces external prior knowledge during the construction of graph data to enhance the physical representation capabilities of graph data. At the same time, to compensate for the shortcomings of physical information graphs, a signal similarity graph is constructed based on signal similarity. In addition, the model designs a differential layer to enhance the node features of the input, thereby alleviating the over-smoothing problem of graph convolutional networks.

 Figure 2 shows the SHAP-LLM joint explanation framework. This method first uses the SHAP algorithm to quantify the contribution of input features to the model’s prediction results and provide a visual explanation of the model. Next, based on the quantification results of the SHAP algorithm, RAG technology is used in combination with an external knowledge base to provide a textual explanation of the model diagnosis and offer certain recommendations.

### 2.1. The PI-Dual-STGCN Fault Diagnosis Framework

#### 2.1.1. Dual-Graph Construction

##### Physical Information Graph Construction

**Figure 3:**
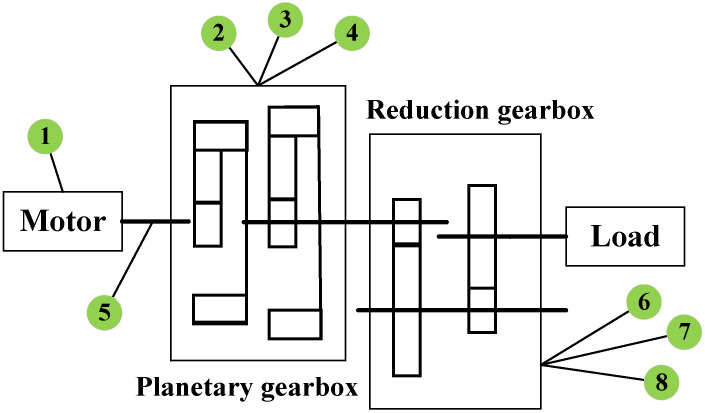
Schematic diagram of gearbox and sensor locations.

Graph construction methods based on physical prior knowledge can effectively overcome the bottleneck of data scarcity. Traditional data-driven methods (such as clustering algorithms) rely on massive samples to accurately capture the relationships between gearbox components, and their graph construction processes have inherent shortcomings such as high computational complexity and limited generalizability. In contrast, by incorporating prior knowledge from mechanical design principles, such as connection topology and motion transmission paths, a physical information graph reflecting the actual structure of the gearbox can be directly constructed. This not only avoids the strong dependence on data volume inherent in traditional methods but also encodes engineering experience, such as assembly constraints between bearings, gears, and shaft systems, into the graph structure. This significantly enhances the physical interpretability of the graph representation while reducing computational resource consumption. A physical information graph was constructed in this study based on the motion transmission path of the gearbox in the target dataset. The dataset used is the Southeast University gearbox dataset (see Section 3 for details), and the schematic graph of the reduction gearbox on the test platform is shown in  Figure 3.

**Figure 4:**
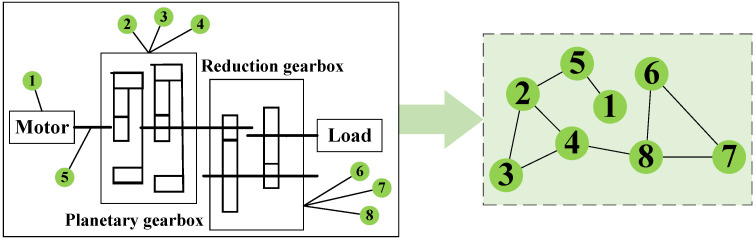
Physical information graph construction.

There are a total of eight channel signals corresponding to the eight columns of data, as follows: column 1 corresponds to the motor vibration signal; columns 2, 3, and 4 correspond to the vibration signals of the planetary gears in the x, y, and z directions, respectively; column 5 corresponds to the motor torque; and columns 6, 7, and 8 correspond to the vibration signals of the reducer in the x, y, and z directions, respectively. A physical information graph is constructed based on the motion transmission path of the gearbox, as shown in  Figure 4. This matrix is learnable during training—it is initialized based on the gearbox’s inherent motion transmission paths. Node 1/5 (motor) and Node 2 (x-direction vibration of the planetary gearbox) form a direct physical transmission path, given that the motor directly drives the planetary gearbox and the x-direction represents the main transmission direction, and thus are interconnected. Nodes 2, 3, and 4 (x/y/z vibrations of the planetary gearbox) reflect the synergistic motion state of the same planetary gearbox, with inherent internal coupling derived from physical priors, and thus are interconnected. Similarly, Nodes 6, 7, and 8 (x/y/z vibrations of the reducer) are coupled as multi-directional response signals of the same reducer, also based on physical prior information, and thus are interconnected. While nodes 4 (planetary gear z-vibration) and 8 (reducer z-vibration) are established because pre-analysis shows their z-direction signals have higher synchronization, non-connected nodes lack significant physical/signal association; thus, no edges are added.

##### Signal Similarity Graph Construction

**Figure 5:**
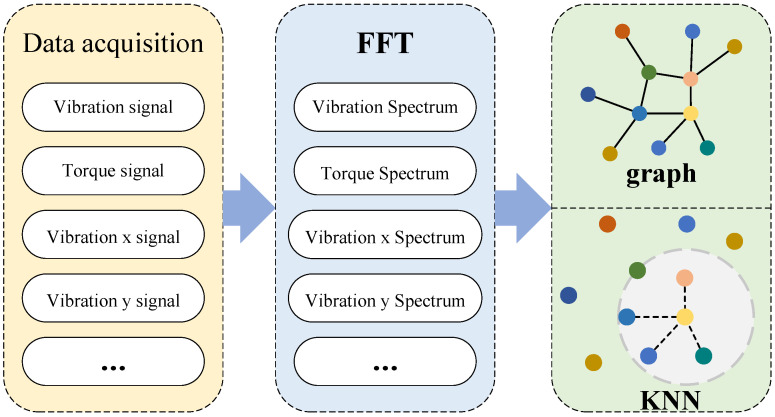
Signal similarity graph construction.

Although graph construction methods based on physical relationships can reveal the intrinsic logic of a system through the spatial characteristics or mechanistic associations of sensor networks, they still have limitations in characterizing the nonlinear dynamic coupling of multidimensional signals under complex operating conditions. In real-world operational scenarios, the time-varying nature of equipment operating states and external disturbances often lead to implicit associations between sensor data that are difficult to characterize using mechanistic models. Such relationships may be hidden within the topological structure or temporal evolution patterns of data distributions. Therefore, it is necessary to construct a signal similarity graph based on signal similarity to assist in training fault diagnosis models using physical information graphs. This study focused on unsupervised clustering algorithms to construct a signal similarity graph that complements the physical information graph, providing data support for subsequent graph neural network models based on dual-graph feature fusion. The graph construction process based on clustering algorithms is shown in  Figure 5.

For the signal similarity graph constructed via KNN on FFT features, the Euclidean distance was selected as the similarity metric and is determined by the characteristics of the FFT features: these features are continuous numerical indicators reflecting the frequency-domain amplitude distribution of signals, and after maximum–minimum normalization, all feature dimensions share a consistent scale. The Euclidean distance can effectively measure the overall amplitude difference between such feature vectors, which aligns with the core demand of characterizing signal similarity (since the frequency-spectrum amplitude distribution is the key basis for judging signal consistency), whereas Manhattan distance only accumulates absolute differences across dimensions (failing to capture the overall spectral difference), and cosine distance focuses on vector direction (neglecting the energy information carried by spectral amplitude). The *k* value in KNN is set to 4: this choice is derived from pre-experiments (in which k=3,4,5,6,7 were tested, and k=4 was found to balance graph connectivity and neighbor relationship distinguishability, avoiding isolated nodes or redundant connections); since this study adopts the standard KNN method to establish hard neighbor connections without kernel function weighting, no kernel bandwidth parameter is involved. In addition, the signal similarity graph in this study is sample-dependent: for each input sample, the FFT features of multiple sensors under the sample are taken as nodes, and a dedicated similarity graph is constructed for the sample via KNN, which adapts to the signal distribution differences of different samples under complex operating conditions and improves the adaptability of the graph structure to the individual features of each sample.

However, this FFT+KNN-based similarity graph has inherent limitations regarding practical industrial applicability: FFT relies on stationary signal assumptions, so its spectral features may blur under variable speed or load fluctuation conditions (reducing similarity measurement accuracy); for early impact-type faults, the weak energy of fault components in the FFT spectrum may lead to indistinct feature differences, making it difficult to capture effective similarity connections.

To clarify the rationality of this strategy, we compare it with typical alternatives aligned with fault mechanisms: Short-Time Fourier Transform (STFT) addresses non-stationarity but faces time-frequency resolution trade-offs, which complicates interpretability for spectral-domain fault analysis; envelope spectra excel at extracting early impact fault features but are sensitive to background noise (limiting stability in noisy industrial environments); order tracking eliminates speed-dependent spectral smearing but requires synchronous rotational speed signals (not available in the SEU dataset used); time-domain statistics are computationally efficient but lack sufficient discriminative power for complex fault signals. In the context of our experimental setup (steady-speed, moderate-load conditions with distinct fault features in the SEU dataset), FFT+KNN balances feature discriminability, computational efficiency, and interpretability consistent with frequency-domain fault mechanisms and thus was selected as the construction strategy.

The k-nearest neighbor (KNN) method is a commonly used method for constructing graphical data that can effectively establish the mutual relationships between nodes in the original signal. Therefore, the KNN algorithm was applied in this study to construct graphical data for multi-sensor signals.

Step 1: To accelerate model convergence, maximum–minimum normalization is used to uniformly standardize the data. The standardization process is as follows:

(1) $$X_{m,j}=\frac{x_{m,i}-x_{\min}}{x_{\max}-x_{\min}}$$

After standardization, the data is divided into subsets with an expected length, and the resulting subsets are shown in the equation.

(2) $$\left\{\begin{array}{c} n=\left\lfloor \frac{L}{d}\right\rfloor ,\\ H_{m}=\left(X_{m,1},X_{m,2},\ldots ,X_{m,n}\right) \end{array}\right.$$

In the formula, $H_{m}$ is the subsample set of the signal; *d* is the step size of the split signal; and *n* is the number of subsamples.

Step 2: In order to enable the model to recognize sensitive features contained in the signal, a fast Fourier transform was performed on each data point in the subsample in this study. The new samples resulting from the transform are nodes in the graphical data. Subsequently, corresponding labels are assigned to each sample.

(3) $$\left\{\begin{array}{c} {\hat{x}}_{m,i}=\mathrm{FFT}\left(X_{m,i}\right)\\ D_{m}=\left[\left({\hat{x}}_{m,1},y_{m,1}\right),\left({\hat{x}}_{m,2},y_{m,2}\right),\ldots ,\left({\hat{x}}_{m,n},y_{m,n}\right)\right] \end{array}\right.$$

Step 3: After obtaining the graph data nodes, the KNN algorithm is used to construct edges, calculate the Euclidean distance between nodes, select the k nodes closest to the central node as the neighboring nodes of the central node, and form edges between each pair of closely related nodes. The data association graph is obtained by finding all the neighbors of each node and repeating the above method to establish edges between neighbors. Each node’s neighbors are obtained using the KNN algorithm, as expressed below.

(4) $$Ne\left(x_{i}\right)=\mathrm{KNN}\left(k,x_{i},D_{m}\right)$$

In the formula, $\mathrm{KNN}(k,x_{i},D_{m})$ represents the first *k* neighbors of node $x_{i}$ in set $D_{m}$; $Ne(x_{i})$ represents the neighbors of node $x_{i}$.

Step 4: After obtaining the neighbors of each node, the weights of the edges can be calculated based on the Gaussian kernel function using the following equation.

(5) $$w_{i,j}=\exp\left(-\frac{∥x_{i}-x_{j}{∥}^{2}}{2\zeta^{2}}\right),\;x_{j}\in Ne\left(x_{i}\right)$$

In the formula, $w_{i,j}$ represents the weight of the edge formed between node $x_{i}$ and node $x_{j}$, and ζ represents the bandwidth of the Gaussian kernel.

Step 5: Repeat the above method for each sensor signal to obtain a multi-source signal graph dataset $G=\{G_{1},G_{2},\ldots ,G_{m}\}$.

A dual-graph fusion architecture based on physical topology and signal similarity achieves efficient and robust feature relationship modeling through the synergistic optimization of prior knowledge constraints and data-driven learning. The physical information graph explicitly encodes the assembly constraints and power transmission paths between gearbox components, significantly reducing dependence on data volume and computational resource consumption for relationship inference, while the signal similarity graph uses statistical learning to capture nonlinear coupling patterns between vibration signals, addressing the limitations of pure physical connections in representing implicit dynamic associations. The two complement each other to form a joint learning paradigm of “structural constraints + dynamic exploration,” ensuring physical interpretability while enabling the model to focus on uncovering essential association features sensitive to faults.

#### 2.1.2. Differential Layer

Before the model performs spatiotemporal convolution operations, a multi-scale differential mechanism is introduced to address the issue of over-smoothing in graph neural networks. This method explicitly captures the gradient changes in feature variations between neighborhoods of different scales through differential operations, thereby preserving local structural sensitivity while suppressing global smoothing trends. The fused differential features are combined with the initial features through residual connections to form a hierarchical feature representation. This alleviates the homogenization of node features caused by deep propagation and constrains the amplitude of the differential through sparse regularization, enabling the model to focus on key scale change regions and significantly enhancing its ability to perceive the hierarchical evolution of graph data.

To elaborate on the design details of this multi-scale differential layer (a key contribution of this work), the following content covers its over-smoothing alleviation mechanism, associations with existing architectures, and computational overhead analysis.

Standard residual graph convolutional networks (GCNs) mitigate feature degradation by fusing initial node features with propagation-derived features, but they still tend to suffer from over-smoothing (i.e., node feature homogenization) after deep propagation. The core limitation lies in that such fusion only retains feature information, rather than capturing the variation patterns between features. In contrast, the proposed multi-scale differential layer explicitly captures feature variation gradients across different-scale neighborhoods (e.g., 1-hop, 2-hop, 3-hop) via differential operations. This operation preserves local feature sensitivity while suppressing global smoothing trends; meanwhile, sparse regularization is applied to constrain differential amplitudes, thereby focusing on key scale-variation regions. The fused differential features are then combined with initial features through residual connections. This design not only inherits the feature retention advantage of residual structures but also enhances the distinguishability of node features, thus alleviating feature homogenization more effectively than standard residual GCNs.

In terms of associations with existing architectures: The layer draws on the multi-scale feature learning idea of Res2Net, which captures spatial multi-scale features via split-convolution in convolutional neural networks (CNNs). However, this layer adapts the idea to graph-structured scenarios by targeting feature variations across neighborhood scales. It also differs essentially from multi-hop GCNs: multi-hop GCNs expand neighborhood ranges to enhance expressive capability but easily exacerbate over-smoothing, while this layer constrains feature homogenization during multi-scale neighborhood interaction via differential operations, thus compensating for the over-smoothing defect of multi-hop GCNs.

For computational overhead: Compared with standard residual GCNs, this layer introduces mild additional costs from multi-scale neighborhood partitioning and differential calculation. In experiments, the number of scales is set to S=4 (validated as a balanced value for performance and overhead), and the corresponding extra computational complexity is O(S×N×D) (where *N* denotes the number of nodes and *D* denotes the feature dimension), accounting for only  15% of the total computational cost of the GCN layer. This moderate increase in overhead is well justified by the significant improvements in over-smoothing alleviation and overall model performance.

**Figure 6:**
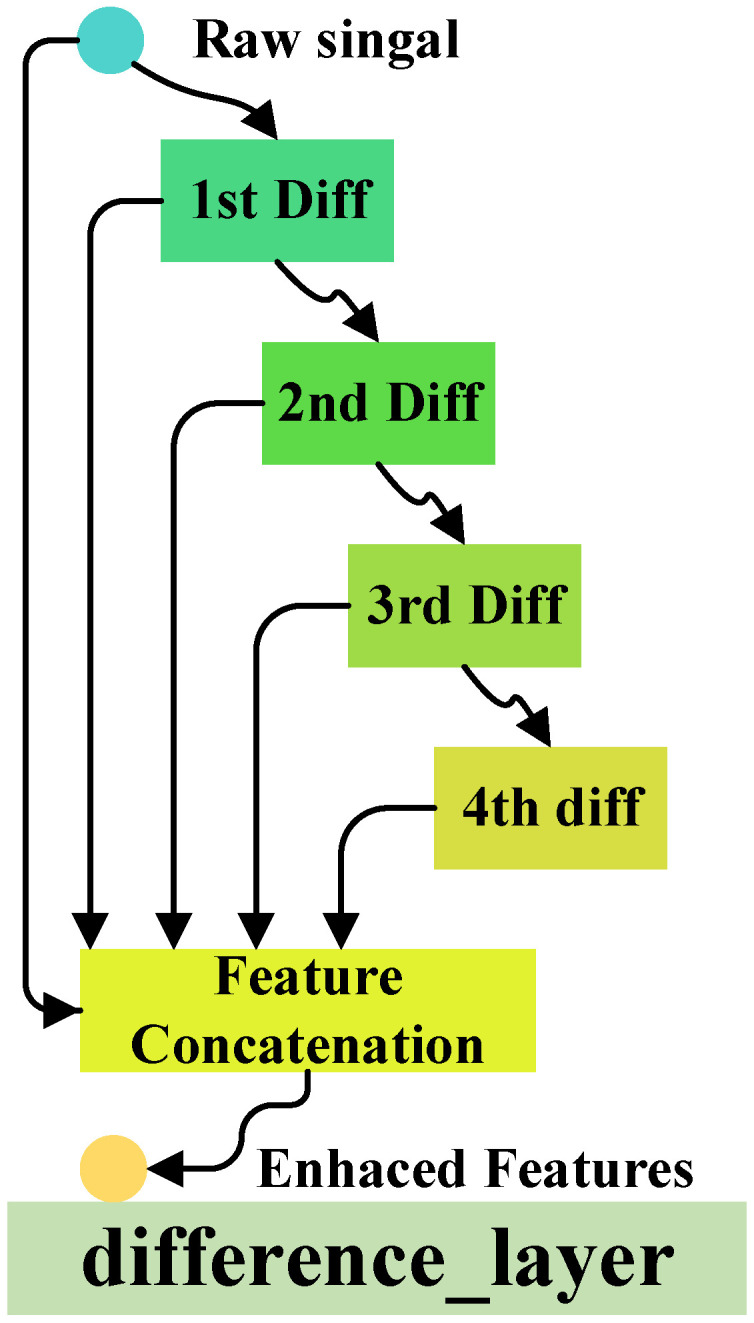
Multiscale differential layers.

The process is illustrated in  Figure 6, with the specific steps as follows.

Step 1: Defining multi-scale channel segmentation

Divide the node features into *K* subsets according to the channel, with each subset corresponding to a different receptive field scale. Let the *l*-th input feature of the layer be $H^{\left(l\right)}\in R^{N\times d}$, and the division formula is

(6) $$H^{\left(l\right)}=\left[H_{1}^{\left(l\right)},H_{2}^{\left(l\right)},\ldots ,H_{K}^{\left(l\right)}\right]$$

In the formula, $H_{K}^{\left(l\right)}\in R^{N\times (d/K)}$.

Reduce computational complexity through grouped convolutions (similar to Res2Net), and force different subsets to learn complementary multi-scale features.

Step 2: Layered residual map convolution formula

Apply graph convolution with adaptive neighborhoods to each subset $H_{K}^{\left(l\right)}$ and introduce cross-scale residual connections:

(7) $$H_{k}^{\left(l+1\right)}=\sigma \left(\underset{\mathrm{Normalized}\;\mathrm{Adjacency}\;\mathrm{Matrix}}{\underset{︸}{{\tilde{D}}_{k}^{-1/2}{\tilde{A}}_{k}{\tilde{D}}_{k}^{-1/2}}}H_{k}^{\left(l\right)}W_{k}^{\left(l\right)}\right)+\sum_{i=1}^{k-1}\alpha_{i,k}H_{i}^{\left(l\right)}$$

In the formula, ${\tilde{A}}_{k}=A+\beta_{k}I$, $\beta_{k}\in R$ controls the self-connection strength (learnable parameter); $\alpha_{i,k}\in R$ is the fusion weight from low-scale features $H_{i}^{\left(l\right)}$ to high-scale features *k*; σ(.) is the activation function.

Step 3: Dynamic multi-scale feature fusion

Use channel attention mechanism to adaptively fuse outputs of different scales

(8) $$a_{k}=\mathrm{Softmax}\left(\frac{1}{N}\sum_{i=1}^{N}H_{k}^{\left(l+1\right)}\left[i,:\right]•\omega_{c}\right)$$

Final output $H^{\left(l+1\right)}=\sum_{k=1}^{K}a_{k}•H_{k}^{\left(l+1\right)}$, where $\omega_{c}\in R^{\left(d/K\right)}$ is the learnable attention vector.

Step 4: Dynamic aggregation of multi-level neighborhoods

Define a multi-order adjacency matrix ${\left\{A^{\left(m\right)}\right\}}_{m=1}^{M}$ ($A^{\left(m\right)}=A^{m}$ representing *m*-hop connections) and select the effective neighborhood through a gating mechanism:

(9) $$G^{\left(m\right)}=\mathrm{Sigmoid}\left(H^{\left(l\right)}W_{g}^{\left(m\right)}\right)$$

(10) $${\hat{A}}^{\left(m\right)}=G^{\left(m\right)}⊙A^{\left(m\right)}$$

Formula for aggregating features of each level:

(11) $$H_{m}^{\left(l+1\right)}=\sigma \left({\tilde{D}}_{m}^{-1/2}{\hat{A}}^{\left(m\right)}{\tilde{D}}_{m}^{-1/2}H^{\left(l\right)}W^{\left(m\right)}\right)$$

Finally, by splicing and merging multi-level features:

(12) $$H^{\left(l+1\right)}=\mathrm{Concat}\left(H_{1}^{\left(l+1\right)},H_{2}^{\left(l+2\right)},\ldots ,H_{M}^{\left(l+1\right)}\right)$$

### 2.2. The SHAP-LLM Joint Explanation Framework

#### 2.2.1. SHAP Interpretation Algorithm and Comprehensive Evaluation Framework

To address the characteristics of deep learning models, a customized model interpreter based on the SHAP library’s Deep Explainer was constructed to compute approximate SHAP values for each feature. Specifically, this method integrates the Deep LIFT algorithm with Shapley value theory: First, feature attribution for prediction differences is achieved by propagating backward through the network, comparing neuron activation values with their reference activations (calculated based on background inputs). Second, to eliminate background correlation effects, a hierarchical sampling strategy was designed to ensure equal sampling of each fault category, guaranteeing inter-class consistency in background value calculations.

The background samples for Deep SHAP are exclusively sourced from the model’s training dataset, ensuring consistency in data distribution between the background data and the data used for model training. This approach avoids distribution shifts that could compromise the reliability of interpretability results. The size of the background dataset is determined by two hyperparameters: the total expected background dataset size $N_{b}$ (denoted as args.shap_background_size) and the total number of fault categories *C* (denoted as args.num_class). To achieve a balanced distribution across different categories and mitigate bias caused by class imbalance, the target number of samples per category is calculated as $n_{c}=\left\lceil N_{b}/C\right\rceil$. The ceiling function ensures adequate representation from each category, while the implicit minimum of 1 guarantees at least one sample per category even when $N_{b}<C$.

We implemented a hierarchical sampling strategy to construct the background dataset, with detailed algorithmic steps presented in Algorithm 1. The strategy comprises three sequential phases:

First, we traversed the entire training dataset $D_{\mathrm{train}}$ and established a class-index mapping dictionary. Each key in this dictionary corresponds to a fault category label, and its associated value stores the indices of all training samples belonging to that category. This step organizes the training samples by their respective classes.

Second, we performed class-wise sample selection based on the pre-calculated per-class target $n_{c}$. For each class *c*, if the number of available samples $|I_{c}|$ was greater than or equal to $n_{c}$, we randomly selected exactly $n_{c}$ samples without replacement using the RandomSubset function. Otherwise, we retained all available samples for that class to preserve its representation. The selected indices from all classes are aggregated to form the unified background sample index set $I_{\mathrm{selected}}$.

Third, we loaded the original data corresponding to each index in $I_{\mathrm{selected}}$ and applied preprocessing to meet the spatiotemporal graph model’s input requirements. Specifically, we converted scalar labels to one-dimensional tensors of shape [1], reshaped sensor data to four-dimensional tensors of shape [1,8,513,1] (batch × sensors × timesteps × channels), and adjusted graph adjacency matrices to two-dimensional matrices of shape [8,8] by removing redundant batch dimensions.

This hierarchical sampling strategy ensures the background dataset is both representative and computationally tractable, providing a stable foundation for Deep SHAP to generate faithful explanations for our spatiotemporal graph model’s predictions.
**Algorithm 1** Hierarchical Background Sampling for Deep SHAP on Spatiotemporal Graph Models**Require:** Training dataset $D_{\mathrm{train}}$ with $N_{\mathrm{train}}$ samples
**Require:** Background dataset size $N_{b}\in N^{+}$**Require:** Number of fault classes $C\in N^{+}$**Require:** Random seed s∈Z for reproducibility
**Ensure:** Background dataset $D_{\mathrm{background}}$ for SHAP explanation

 1: Calculate target samples per class: $n_{c}\leftarrow \left\lceil N_{b}/C\right\rceil$

 2: Initialize class_indices as empty dictionary

▹ **Step 1: Organize samples by class** 

 3: **for** 
i=0
** to **
$|D_{\mathrm{train}}|\;-\;1$
** do**

 4:        $y_{i}\leftarrow D_{\mathrm{train}}\left[i\right].\mathrm{label}$

 5:        class_indices$\left[y_{i}\right]\leftarrow$ class_indices$\left[y_{i}\right]\cup \left\{i\right\}$

 6: **end for**

▹ **Step 2: Stratified sampling** 

 7: $I_{\mathrm{selected}}\leftarrow \emptyset$

 8: **for **
c=0
** to **
C−1
** do**

 9:      $I_{c}\leftarrow$ class_indices[c]

10:      **if** $|I_{c}|\;\geq n_{c}$ **then**

11:           $I_{c}^{\mathrm{selected}}\leftarrow \mathrm{RandomSubset}\left(I_{c},n_{c}\right)$

12:     **else**

13:            $I_{c}^{\mathrm{selected}}\leftarrow I_{c}$
▹ Use all available samples

14:      **end if**

15:       $I_{\mathrm{selected}}\leftarrow I_{\mathrm{selected}}\cup I_{c}^{\mathrm{selected}}$

16: **end for**

▹ **Step 3: Load and preprocess samples** 

17: $D_{\mathrm{background}}\leftarrow \left[\;\right]$

18: **for** each index $i\in I_{\mathrm{selected}}$ **do**

19:       $x\leftarrow D_{\mathrm{train}}\left[i\right]$
20:

▹ Preprocess for model input compatibility:

21:       x.label←unsqueeze(x.label,0)

▹ To shape [1]

22:       x.sensor_data ←reshapeto[1,8,513,1]

23:       x.graph←squeeze(x.graph,0)
▹ To shape [8,8]

24:       $D_{\mathrm{background}}.\mathrm{append}\left(x\right)$

25: **end for**

26: **return **
$D_{\mathrm{background}}$


**Figure 7:**
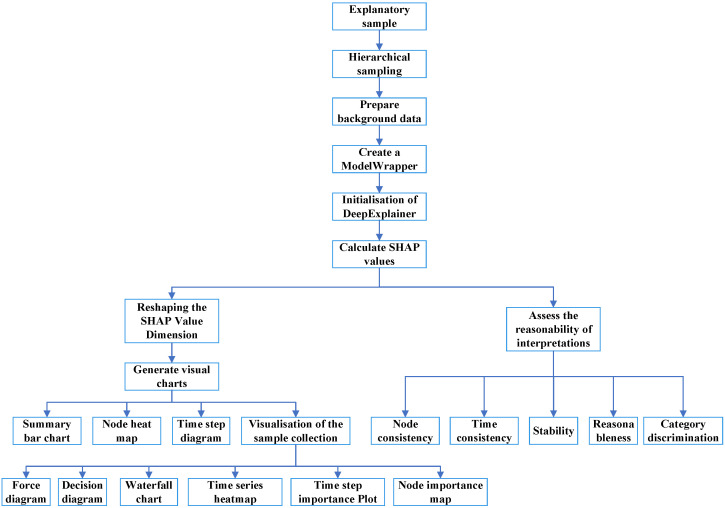
SHAP interpretation method and comprehensive evaluation framework.

As noted in the Introduction, most existing applications of SHAP in fault diagnosis only utilize SHAP to analyze the model’s feature importance (treating SHAP as a tool for model assessment), rather than evaluating the rationality and reliability of the SHAP explanations themselves. To address this gap, the proposed comprehensive evaluation framework—encompassing node importance consistency, time step importance consistency, interpretation stability, interpretation rationality, and category discrimination—is designed to directly target the rationality of SHAP explanations, rather than merely using SHAP as an auxiliary model analysis tool. Based on the above algorithm principles and objective characteristics, this study designed the SHAP interpretation method and comprehensive evaluation framework as shown in Figure 7. These metrics complement existing general explanation quality metrics (e.g., Faithfulness, Robustness) with adjustments tailored to the fault diagnosis scenario: Faithfulness, a widely used general metric, only assesses alignment between explanations and model predictions but overlooks cross-sample explanation consistency—a core requirement for fault diagnosis, where identical fault types should correspond to stable explanation patterns; Robustness focuses on explanation stability under input perturbation conditions but lacks adaptation to the spatiotemporal characteristics (multi-sensor nodes, time-step dimensions) of the spatiotemporal graph model in this study. In contrast, our metrics are customized to both the fault diagnosis task and the model’s spatiotemporal structure, covering node-level/time-step-level consistency (matching the model’s dual-dimensional architecture) and category distinguishability (aligning with the task’s need to identify fault-specific patterns), thereby addressing the scenario-specific limitations of general metrics. For judging the rationality of SHAP explanations, reference ranges for each metric are determined based on experimental results of the target fault diagnosis task: the node importance consistency, time step importance consistency, interpretation stability, and interpretation rationality—each with a value range of (0, 1]—are all considered indicative of reliable SHAP explanations when their values exceed 0.75; category discrimination is deemed reliable when this distance is greater than 1 as this indicates distinct fault-specific patterns.

For the SHAP algorithm used in fault diagnosis explanations in this study, the following characteristics exist:

(1) Node importance should be consistent. When using different samples of the same fault to explain the model prediction logic multiple times, the ranking of feature contributions should be similar, and node importance should remain consistent across different samples.

(2) The importance of time steps should be consistent. The PI-Dual-STGCN model simultaneously captures spatiotemporal dependencies. Under constant operating conditions, the same fault exhibits consistency in its frequency components. Therefore, the time-dimensional SHAP values reflecting the model’s ability to locate fault-sensitive frequency bands should exhibit consistency.

(3) The interpretation of the same fault sample should be stable. Changes in background samples should not cause drastic changes in explanation results; multiple explanations of the same fault sample should produce similar results.

(4) Explanations should be consistent with model behavior. The most important features identified by SHAP should be the primary motivators for the model’s diagnosis, and these features should significantly influence the model’s prediction confidence.

(5) Different categories should exhibit distinguishability. Different fault samples should have distinct key feature patterns, and explanations should reflect the specificity of different faults.

The comprehensive evaluation framework includes node importance consistency indicators, time step importance consistency indicators, interpretation stability indicators, interpretation rationality indicators, and category discrimination indicators.

(1) Node importance consistency indicators.

In the field of fault diagnosis, samples of the same fault type should have similar distributions of important sensor nodes. The node importance consistency index aims to quantify the consistency of SHAP explanations in identifying key nodes across different samples. If the model explanation is reasonable, samples of the same fault type should exhibit similar important node patterns. Let the number of samples be *N*, the number of nodes be *M*, and the sum of the absolute SHAP values of the *i* node in the *j* sample be

(13) $$v_{ij}=\sum_{t=1}^{T}\left|SHAP_{ijt}\right|$$

where *T* is the time step.

Node importance matrix $V\in R^{N\times M}$, where $V_{i,j}=v_{ij}$.

The standard deviation of the importance of nodes *j* is

(14) $$\sigma_{j}=\sqrt{\frac{1}{N-1}\sum_{i=1}^{N}{\left(v_{ij}-\mu_{j}\right)}^{2}}$$

where $\mu_{j}=\frac{1}{N}\sum_{i=1}^{N}v_{ij}$.

The node importance consistency index is defined as

(15) $$NodeConsistency=\frac{1}{1+\frac{1}{M}\sum_{j=1}^{M}\sigma_{j}}$$

The value range of this indicator is (0, 1], with higher values indicating better consistency.

(2) Time step importance consistency indicators.

Faults typically exhibit specific frequency domain evolution patterns, and a reasonable time step importance distribution should reflect this characteristic. This metric evaluates the consistency of SHAP values across time dimensions, ensuring that the key time points identified by the model remain consistent across samples of the same fault type.

Let the sum of the absolute SHAP values at the *t* time step for the *i* sample be

(16) $$u_{it}=\sum_{j=1}^{M}\left|SHAP_{ijt}\right|$$

Time step importance matrix $U\in R^{N\times T}$, where $U_{i,t}=u_{it}$.

The importance of time steps’ standard deviation is

(17) $$\sigma_{t}=\sqrt{\frac{1}{N-1}\sum_{i=1}^{N}{\left(u_{ij}-\mu_{j}\right)}^{2}}$$

The time step importance consistency index is defined as

(18) $$TimeConsistency=\frac{1}{1+\frac{1}{T}\sum_{t=1}^{T}\sigma_{t}}$$

The value range of this indicator is (0, 1], with higher values indicating better consistency.

(3) Interpretation stability indicators.

A reliable model explanation should be stable, meaning that it produces similar results when run multiple times. This metric assesses the stability of the explanation method by repeatedly calculating SHAP values and comparing the similarity of the results.

Performing *R* SHAP interpretation on the sample *i* leads to a sequence of node importance vectors $v_{i}^{\left(1\right)},v_{i}^{\left(2\right)},\ldots ,v_{i}^{\left(R\right)}$.

The cosine similarity between any two interpretations *r* and *s* is calculated:

(19) $$sim_{rs}^{\left(i\right)}=\frac{v_{i}^{\left(r\right)}\cdot v_{i}^{\left(s\right)}}{∥v_{i}^{\left(r\right)}∥\cdot ∥v_{i}^{\left(s\right)}∥}$$

The stability score for the sample *i* is

(20) $$stability_{i}=\frac{2}{R(R-1)}\sum_{r=1}^{R-1}\sum_{s=r+1}^{R}sim_{rs}^{\left(i\right)}$$

The overall stability index is the average stability of all samples:

(21) $$Stability=\frac{1}{N}\sum_{i=1}^{N}stability_{i}$$

The value range of this indicator is (0, 1], with higher values indicating better consistency.

(4) Interpretation rationality indicators.

This metric validates the validity of SHAP explanations by perturbing key features and observing changes in predictions. If the important features identified by SHAP are indeed critical, the prediction probability should change significantly after perturbation.

The original predicted probability of the sample *i* is $p_{i}$. According to SHAP interpretation, the most important node $j^{*}=\arg\max_{j}v_{ij}$ is found. A perturbation sample is created, and the node $j^{*}$ data are set to zero: $x_{i}^{\mathrm{pert}}=x_{i}$ but $x_{i,j^{*},t}=0,\forall t$.

The predicted probability $p_{i}^{\prime }$ after perturbation is calculated. The rationality score is defined as

(22) $$Plausibility_{i}=\frac{p_{i}-p_{i}^{\prime }}{p_{i}}$$

The overall rationality index is

(23) $$Plausibility=\frac{1}{N}\sum_{i=1}^{N}plausibility_{i}$$

The value range of this indicator is (0, 1], with higher values indicating better consistency.

(5) Category discrimination indicators.

Different fault types should have different explanation patterns. This indicator quantifies the distinguishability of SHAP explanations across different fault categories, ensuring that the model can distinguish the unique characteristics of different faults.

The average node importance vector for each category *c* is calculated:

(24) $$w_{c}=\frac{1}{n_{c}}\sum_{i\in I_{c}}v_{i}$$

where $I_{c}$ is the sample index set of category *c*, and $n_{c}$ is the number of samples in category *c*.

The Euclidean distance between category *c* and category *k* is

(25) $$d_{ck}={∥w_{c}-w_{k}∥}_{2}$$

The category discrimination index is defined as the average value of the distance between all categories:

(26) $$ClassDistance=\frac{1}{C(C-1)}\sum_{c=1}^{C}\sum_{k\neq c}d_{ck}$$

where *C* is the number of categories. The value range of this indicator is [0,∞), and the larger the value, the greater the difference in interpretation between different categories.

The SHAP explanation rationality evaluation index system proposed in this paper evaluates the quality of algorithm explanations from multiple dimensions, providing a scientific explanation verification method for SHAP. This framework is not only applicable to the PI-Dual-STGCN model in this study but can also be extended to other deep learning-based fault diagnosis models, providing theoretical support and practical guidance for the application of explainable AI in industrial fields.

#### 2.2.2. RAG-Based LLM Explanation Method

An external knowledge base for fault diagnosis was constructed in this study, and a fault diagnosis explanation LLM based on RAG retrieval enhancement technology was designed, combining node quantification information from the SHAP algorithm.

To support the fault diagnosis and interpretation system, we constructed a structured external knowledge base comprising five key components, each serving specific functions in the diagnostic process and explanation generation.

The *Common Gearbox Failures and Related Insights* module provides detailed engineering knowledge concerning five failure states covered in the dataset (tooth surface defects, tooth loss, root cracks, tooth surface wear, and healthy operation), including their manifestations, impacts, diagnostic characteristics, and practical maintenance countermeasures. This knowledge enables the system to ground its fault analysis in established engineering principles and provide maintenance recommendations consistent with industrial best practices.

The *Sensor Placement* module documents the physical configuration of eight sensors in the experimental setup, explicitly mapping each sensor node (0–7) to its monitoring function and physical location within the gearbox system. This mapping allows the system to translate abstract node importance scores from SHAP into concrete, sensor-specific insights that maintenance personnel can directly relate to physical components.

The *Fault Diagnosis Model Performance Metrics* module defines standard evaluation criteria including accuracy, precision, recall, and F1-score, along with their mathematical interpretations and practical significance. This component provides the conceptual framework for assessing diagnostic model effectiveness and generating quantitative performance analyses.

The *SHAP Reliability Assessment Criteria* module establishes quantitative thresholds for evaluating explanation quality, with four key indicators—node consistency, temporal consistency, stability, and plausibility—each requiring a minimum threshold of 0.75 to ensure high-quality explanations. Additionally, class distinctness is maintained at a threshold of 1.2 to ensure clear differentiation between fault types. These criteria serve as objective benchmarks for validating the trustworthiness and reliability of SHAP-based explanations.

The *Principles for Explaining Fault Diagnosis Models* module outlines four fundamental requirements for effective explanations: understandability, consistency with domain knowledge, reliability, and actionability. These principles guide the explanation generation process to ensure outputs are both technically sound and practically useful for on-site decision-making.

All knowledge components are organized with clear topic delimiters (“[[”), facilitating efficient retrieval through the RAG system. This knowledge base enables the RAG-enhanced LLM to retrieve relevant domain knowledge and generate explanations that bridge AI diagnostics with engineering understanding.

This study employed a locally deployed DeepSeek-8B model as the foundational large language model (LLM), utilizing RAG technology for diagnostic report generation. Specifically, the system first processes SHAP explanations and model performance metrics as queries, then leverages a vector database to retrieve the top three most relevant text segments from external knowledge repositories. Subsequently, the retrieved content, original query information, and task prompt are synthesized into an augmented prompt. Finally, the large language model generates accurate, traceable, and contextually coherent responses based on this prompt, thereby effectively mitigating model hallucinations and enhancing the reliability and timeliness of answers.

To improve the determinism of the LLM’s outputs, two design constraints are incorporated into the generation pipeline: First, augmented prompts include explicit, task-aligned guidelines that define the content scope and structural norms of the target report, reducing unstructured or deviated content generation. Second, the RAG module provides grounded factual fragments (retrieved from external knowledge repositories) as the factual basis for report content, limiting the model’s reliance on parametric knowledge and thus mitigating unsubstantiated output. These measures collectively enhance the consistency of the model’s outputs for identical input queries across multiple inference runs. To further enhance the determinism of the model’s outputs, the inference parameter ‘temperature’ of the DeepSeek-8B model is set to 0.3: in LLM inference, a lower ‘temperature’ value reduces the randomness of token sampling, thereby promoting more consistent and predictable outputs for identical inputs.

The fault diagnosis model explanation report designed in this study is divided into a main report on model reliability and a sub-report on detailed fault analysis. The main report on model reliability is used to evaluate the performance of the fault diagnosis model and the reliability of the SHAP explanation algorithm, which involves information from the knowledge base and the actual evaluation metric values of the model. Information from the knowledge base is input into the large model via RAG technology, while the model’s actual real-time evaluation metric values are achieved through programs designed within the LLM explanation function and prompt. The main report enhances engineers’ confidence in the diagnostic model’s prediction results and the consistency between SHAP explanation results and the model’s actual predictive behavior by analyzing the model’s diagnostic performance and the reliability of the SHAP explanation algorithm. These measures aim to strengthen engineers’ confidence in using the model’s diagnostic results and explanation results. To enhance the readability and visual appeal of the report, key metrics are highlighted in the main report, along with key visualization charts, and links to sub-reports are provided. Sub-reports are primarily used for detailed diagnostic analysis of fault data, presenting content such as fault types, fault graphs, node contribution data and visualization charts, model decision-making criteria, and detailed analysis reports of fault samples.

**Figure 8:**
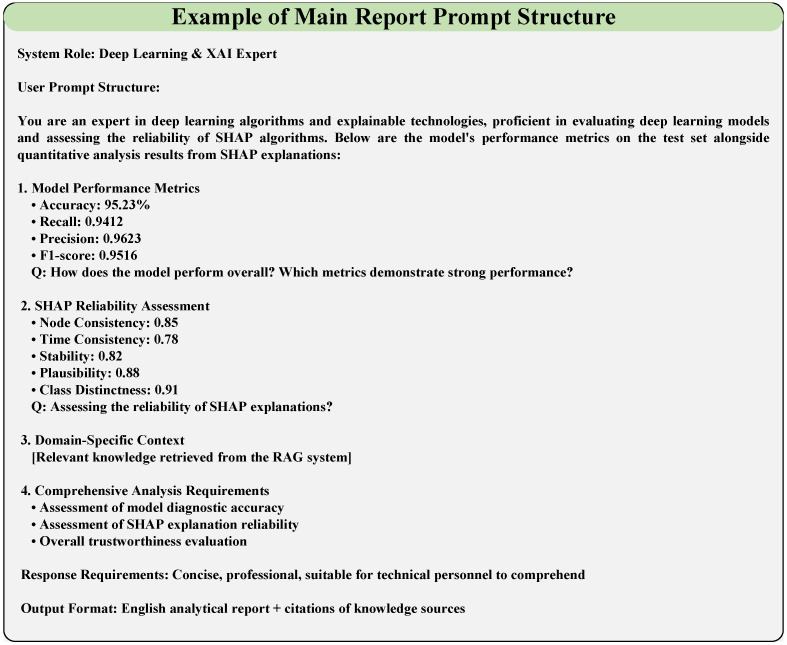
The structured prompt frameworks for the main report.

**Figure 9:**
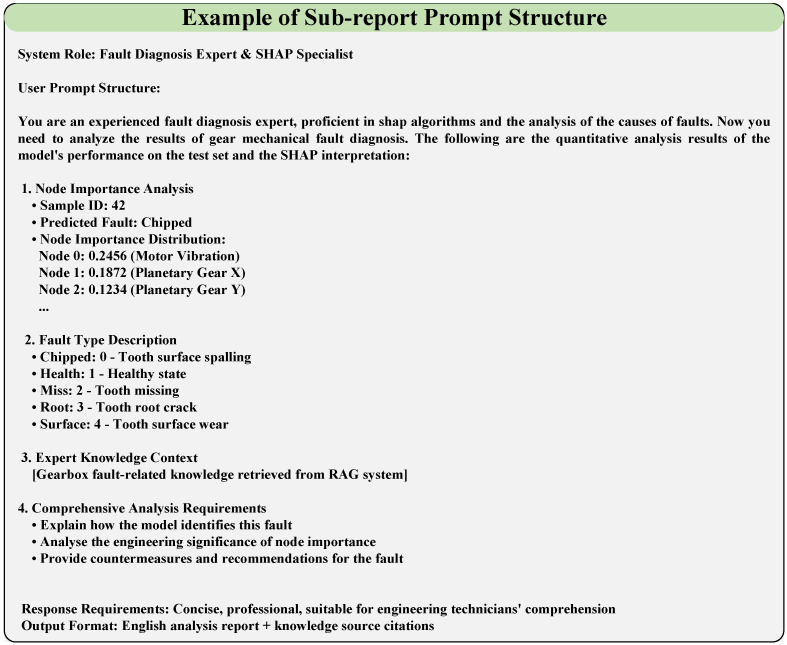
The structured prompt frameworks for the sub-report.

The structured prompt frameworks for the main report and sub-report (illustrated in Figure 8 and Figure 9, respectively) are designed to guide the LLM in generating targeted, engineering-friendly diagnostic analyses, with their core components and concrete content derived from the task requirements and experimental results.

For the main report (overall fault diagnosis model analysis, Figure 8), the prompt framework is constructed with a two-tier structure (system role definition + user task content) and incorporates four core information modules. The system role is defined as “an expert in deep learning algorithms and explainable technologies, proficient in evaluating deep learning models and assessing the reliability of SHAP algorithms”.

For the sub-report (fine-grained individual sample fault analysis, Figure 9), the prompt framework retains the two-tier structure (system role + user task content) but is tailored for component-level detailed analysis, with its core content focusing on node importance and fault-specific interpretation. The system role is redefined as "an experienced fault diagnosis expert, proficient in SHAP algorithms and the analysis of the causes of faults" to align with the fine-grained analysis task.

## 3. Experimental Verification of Fault Diagnosis Algorithms

To comprehensively evaluate the performance of the proposed PI-Dual-STGCN model for fault diagnosis tasks, comparative experiments against several representative state-of-the-art methods were conducted in this study. Given that the proposed PI-Dual-STGCN model incorporates multiple attributes such as temporal reasoning, graph neural networks, and multi-graph structures, the selected comparison methods span diverse modeling paradigms to validate our model’s capabilities. These methods include

(1) Methods based on time-series modeling

MCN-LSTM [33]: This method combines MCNN and the long short-term memory network LSTM, excelling at capturing the multi-scale temporal features and long-term dependencies of signals. It is a classic method for handling time-series fault diagnosis.

(2) GNN-based fault diagnosis/prediction methods

GAT [34]: The Graph Attention Network is one of the foundational models of graph neural networks. It dynamically aggregates neighbor information by learning attention weights between nodes, serving as a benchmark for evaluating graph modeling capabilities.

GGCN [35]: The Gate Graph Convolutional Network is used for multi-sensor signal fusion in remaining useful life prediction, demonstrating the application of a GCN in industrial prediction tasks.

HAGCN [36]: The Hierarchical Attention Graph Convolutional Network is also used for multi-sensor signal fusion and remaining useful life prediction, introducing a hierarchical attention mechanism.

MNQGN [37]: The Multi-Scale Neighborhood Query Graph Convolutional Network focuses on mining interactions between neighboring nodes at different scales based on a fixed graph structure used for multi-defect localization in composite materials.

MRF-GCN [38]: Multi-Receptive Field Graph Convolutional Networks construct multiple receptive fields (receiving domains) to aggregate information from different neighbor ranges, aiming to address the limitations of a single receptive field, applied to machine fault diagnosis.

(3) Multi-graph-based methods

AM-GCN [39]: The core innovation of adaptive multi-channel graph convolutional networks lies in utilizing node features, topological structures, and their higher-order combinations to adaptively construct and fuse multiple graph channels.

### 3.1. Experimental Setup and Evaluation Criteria

**Figure 10:**
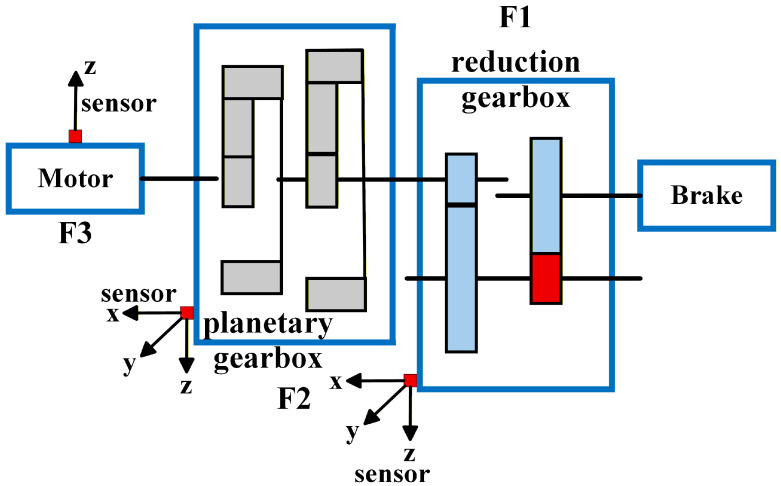
Schematic diagram of the experimental platform.

This experiment used the Southeast University gearbox dataset [40]. It should be noted that the Southeast University gearbox dataset is a well-recognized public benchmark dataset, and the dynamic behavior differences of this system in various fault states have been comprehensively characterized and validated in the dataset’s original publication [40]. The dataset description is as follows: The experimental platform, as shown in Figure 10, consists of six main components: the motor controller, the motor (F3), the planetary gearbox (F2), the reduction gearbox (F1), the load, and the load controller. Seven vibration sensors of model 608A11 were installed to collect vibration data along the x-, y-, and z-axes of the planetary gearbox and reduction gearbox, as well as the z-axis direction of the motor. The sampling frequency was 5120 Hz. On this experimental platform, vibration data was simulated and collected under different operating conditions and gear states, including tooth breakage, healthy or missing teeth, root cracks, and gear surface wear, with a balanced distribution of fault categories. Gears in different fault states were pre-manufactured. Variable speed can be achieved through the motor controller, while load changes can be achieved through the load controller. Additionally, the faulty gears are installed inside the reduction gearbox.

In this experiment, all 8 channel signals were used, and the original data was segmented using the sliding window method, with a window size of 1024, a sliding step size of 1024, and no overlap between samples, resulting in 5115 samples. A fast Fourier transform (FFT) was performed on each sample, converting 1024 points in the time domain to 513 points in the frequency domain. The data was normalized using Min–Max normalization. To ensure that the proportions of each fault category were roughly consistent, a stratified random sampling method was used to select 80% of the samples as the training set and 20% as the test set. After dividing the training set and test set, the graph data was constructed according to Section 2.

The PI-Dual-STGCN algorithm was implemented using Python 3.8 and PyTorch 1.9, with all experiments conducted on the hardware/software environment detailed in Table 1. Training the model for 80 epochs required approximately 2.5 h. Hyperparameter selection followed a combination of empirical testing and systematic search. The number of difference layers was systematically evaluated over {1, 2, 3, 4, 5, 6} via grid search. The node feature dimension (set to 5 in the final model) is derived from enhanced data after multi-scale differential processing, following the rule that “node feature dimension = number of differential dimensions + 1”. Other parameters were determined through empirical experimentation: the learning rate ($1\times 10^{-4}$) and weight decay ($1\times 10^{-5}$) were selected after testing values in the ranges $1\times 10^{-5}$ to $1\times 10^{-3}$ and $1\times 10^{-6}$ to $1\times 10^{-4}$, respectively. The batch size was set to 64 as a balance between memory constraints and training stability, considering options of 32, 64, and 128. Training was conducted for 80 epochs, which ensured convergence based on validation loss monitoring. All selections were validated by the final model performance on the validation set.

To ensure a fair and rigorous comparison, all baseline methods were evaluated under identical conditions. Their input representation (8-channel signals), window size (1024 sampling points), sliding step size (1024 sampling points), FFT dimension (513 points), normalization method (Min–Max), and data split (80% training/20% testing via stratified sampling) were kept identical to those of the proposed PI-Dual-STGCN. The key hyperparameters (e.g., learning rate, batch size) for baseline models were determined following this protocol: when explicit settings were provided in their original publications, those were adopted; otherwise, a limited grid search was performed using the same parameter ranges as for our proposed method (e.g., learning rate from $1\times 10^{-5}$ to $1\times 10^{-3}$). Training stopping criteria followed the original settings of each method, with early stopping (patience = 10 epochs) applied only to methods without a specified rule. To account for randomness in training, each baseline method was evaluated over five independent runs. The average results and corresponding standard deviations from these runs serve as the comparison standard.

Evaluation metrics: Accuracy, precision, recall, and F1-score are used as the primary evaluation metrics to comprehensively measure the classification performance of the model. The calculation formulas are as follows:

(27) $$\mathrm{Accuracy}=\frac{\mathrm{TP}+\mathrm{TN}}{\mathrm{TP}+\mathrm{TN}+\mathrm{FP}+\mathrm{FN}}$$

(28) $$\mathrm{Precision}=\frac{\mathrm{TP}}{\mathrm{TP}+\mathrm{FP}}$$

(29) $$\mathrm{Recall}=\frac{\mathrm{TP}}{\mathrm{TP}+\mathrm{FN}}$$

(30) $$F1\text{-}\mathrm{Score}=\frac{2\times (\mathrm{Precision}\times \mathrm{Recall})}{\mathrm{Precision}+\mathrm{Recall}}$$

where TP is a true positive example, TN is a true negative example, FP is a false positive example, and FN is a false negative example.

### 3.2. Results and Analysis

To ensure the reliability and stability of the experimental results, each model was evaluated over five independent runs (the hyperparameters of all models were determined through grid search in advance). The final evaluation criteria for model performance consist of the mean values and corresponding standard deviations (denoted as mean ± SD) of four key metrics: accuracy (Acc), precision (P), recall (R), and F1-score (F1).

**Figure 11:**
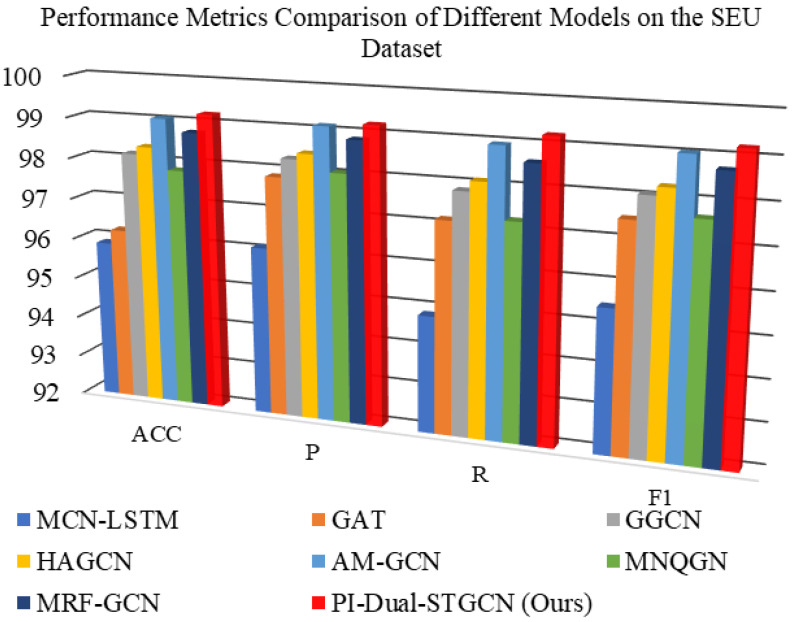
Performance of different models on the SEU dataset.

Table 2 presents the comprehensive performance comparison of different models in fault diagnosis tasks based on the SEU dataset, where both mean metrics and their standard deviations are reported to ensure result completeness. Correspondingly, Figure 11 provides the visual characterization of the mean performance (derived from five independent runs) of various models across four core evaluation metrics on the SEU dataset. This figure intuitively demonstrates the performance gaps among different models. Such a visual presentation complements the tabular data, making the superiority of the proposed model more straightforward to perceive.

As can be clearly observed from Table 2 and Figure 11, all models perform well on the SEU dataset, but the PI-Dual-STGCN model proposed in this paper significantly outperforms the comparison methods across all evaluation metrics. Its F1-score reaches 99.22% (with a standard deviation of only 0.08), which is 0.19 percentage points higher than the next-best model, AM-GCN (F1 = 99.03%, SD = 0.17)—this not only reflects superior diagnostic accuracy but also outstanding performance stability across repeated experiments. This fully demonstrates the effectiveness and superiority of PI-Dual-STGCN in fault diagnosis tasks.

Structural design differences among various algorithms lead to significant variations in both performance and stability. The temporal baseline model MCN-LSTM, which does not model sensor spatial correlations, achieves the lowest recall rate of 94.85% accompanied by a relatively large standard deviation (0.67), revealing the inherent limitations of pure temporal architectures in multimodal diagnosis (insufficient spatial feature modeling also leads to poor result reproducibility). GAT relies on data-driven adaptive weight allocation, and the significant gap between its 97.85% precision rate and 97.15% recall rate reflects the sensitivity of single-technology learning to feature noise; meanwhile, its metrics exhibit moderate fluctuations (e.g., SD = 0.47 for precision), implying that data-driven attention weights are prone to instability under noise interference conditions.

MNQGN is constrained by a static neighbor query mechanism, with a recall rate of 97.25% lagging behind an accuracy rate of 98.05%; its relatively large standard deviation (0.46 for recall) further confirms that rigid graph structures struggle to capture dynamic fault propagation, leading to unstable experimental results. Although the GGCN and HAGCN introduce feature fusion mechanisms, they are constrained by single-graph limitations, with F1-scores of 98.07% and 98.27% indicating bottlenecks in modeling complex systems; their standard deviations (0.37 and 0.30 for F1, respectively) are notably higher than that of PI-Dual-STGCN, revealing that single-graph architectures still face stability bottlenecks when handling multi-source fault information.

MRF-GCN enhances neighborhood aggregation capability to a recall rate of 98.60% through a multi-receptive field design, but due to the absence of physical prior knowledge, its overall performance of 98.72% still lags behind this method; its standard deviation (0.26 for F1) is slightly larger than that of AM-GCN, suggesting that multi-receptive field designs without physical constraints still have limited stability. The strongest baseline, AM-GCN, validates the effectiveness of multi-graph fusion, but its completely data-driven graph construction results in a recall rate of 98.95% (which lags behind this method); while its performance is close to the proposed model, its standard deviation (0.17 for F1) is nearly twice that of PI-Dual-STGCN, highlighting the necessity of incorporating physical knowledge to simultaneously improve diagnostic accuracy and result stability.

### 3.3. Ablation Experiment

To validate the effectiveness of each core component of PI-Dual-STGCN, rigorous ablation experiments were designed in this study. All experiments were conducted under the same dataset and training conditions, with the results shown in Table 3.

The results indicate that when only the signal association graph is used (removing the physical information graph, “w/o PI Graph”), the model’s F1-score decreases by 0.61 percentage points to 98.61% (with P = 98.70% ± 0.20, R = 98.52% ± 0.22)—the slight gap between precision and recall reflects that lacking physical topology constraints weakens the model’s ability to balance positive/negative sample prediction, while the increased standard deviation (vs. the full model) indicates reduced stability. When relying solely on the physical information graph (removing the signal association graph, “w/o Signal Graph”), the F1-score decreases by 0.54 percentage points to 98.68% (with P = 98.75% ± 0.18, R = 98.63% ± 0.20): this implies data-driven signal correlations are necessary to compensate for fixed physical topology’s limitations, and the stable (but lower) performance confirms physical priors still provide basic prediction rationality.

When a fully connected graph replaced the dual graph (“Single-Graph”), model performance plummeted by 1.31% (F1 = 97.91% ± 0.33, P = 98.00% ± 0.31, R = 97.82% ± 0.35): the large performance drop and higher standard deviations (the least stable among all variants) confirm that physical topology constraints and data-driven associations are complementary—the former provides physical rationality, the latter adapts to data fluctuations. Thus, the physical information-guided dual graph mechanism is a key innovation.

When the multi-scale differential (MS-Diff) layer is removed (“w/o MS-Diff”), the F1 score decreases by 0.36 percentage points to 98.86% (with P = 98.90% ± 0.14, R = 98.82% ± 0.16). The small but significant drop (paired with stable standard deviations) indicates MS-Diff effectively captures fine-grained fault features across scales without introducing instability, validating its role in enhancing feature representation.

To directly characterize over-smoothing (beyond classification performance) and evaluate representation quality, we introduce three quantitative metrics (results in Table 4):

- Average Inter-layer Node Embedding Similarity: Mean cosine similarity of node features between consecutive model layers (higher values indicate more severe feature homogenization, i.e., over-smoothing);
- Intra-/Inter-Class Distance Ratio: Ratio of mean intra-class feature distance to mean inter-class feature distance (lower values reflect better separability of fault class representations);
- SHAP Score Stability: Standard deviation of SHAP feature importance scores across 100 test samples (smaller values correspond to more stable model decision explanations).

Combined with Table 4, we can explicitly observe how removing the MS-Diff layer degrades representation separability and explanation stability:

1. The average inter-layer node embedding similarity rises from 0.63 (full model) to 0.71 (an increase of 12.7%), which directly indicates more severe feature homogenization—the core manifestation of over-smoothing.
2. The intra-/inter-class distance ratio increases from 0.47 to 0.51 (an 8.2% rise), meaning the separability of feature representations between different fault classes is weakened.
3. The SHAP score standard deviation expands from 0.08 to 0.10 (a 25.0% growth), reflecting that the model’s decision explanation logic becomes less consistent across test samples.

These results validate that the MS-Diff layer not only preserves classification performance but also mitigates over-smoothing, maintains representation separability, and ensures explanation stability.

## 4. Experimental Verification of Model Interpretability Solutions

**Figure 12:**
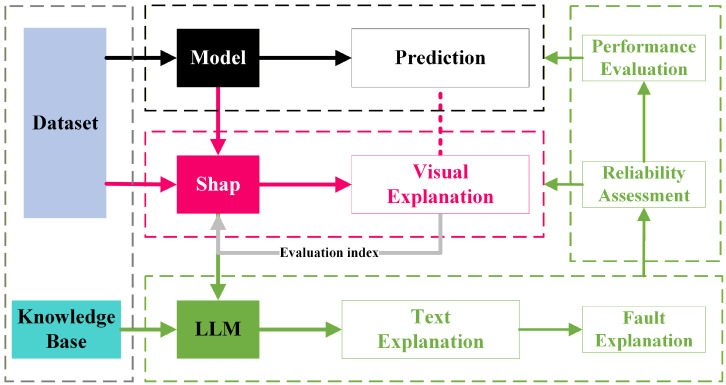
Model interpretability scheme.

The model interpretability scheme design, as shown in Figure 12, is a joint SHAP-LLM explanation framework. This framework first uses the SHAP algorithm based on the Deep Explainer interpreter to quantify the contribution of input features to the prediction results of the fault diagnosis model. It then uses quantitative information visualization to achieve visual interpretation of the model for five types of fault diagnosis logic. Based on the SHAP-quantified information, and in combination with an external knowledge base using RAG retrieval enhancement technology, the framework leverages the powerful reasoning capabilities of large models to provide natural language explanations of model performance, the reliability of SHAP explanations, and prediction logic, along with relevant evaluation results and recommendations.

### 4.1. Visual Interpretation of Model Diagnostics Based on SHAP

**Figure 13:**
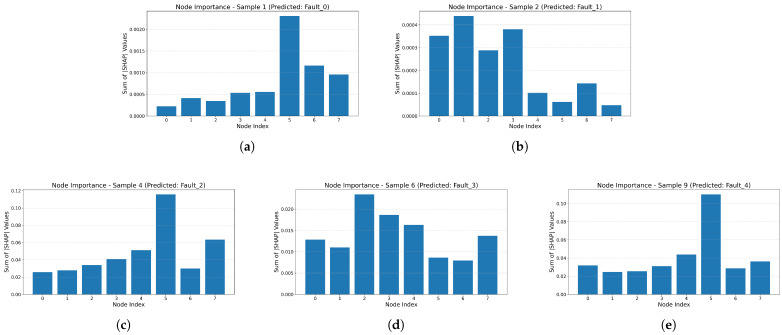
The importance of nodes with different faults. (**a**) Chipped. (**b**) Health. (**c**) Miss. (**d**) Root. (**e**) Surface.

The visual interpretation results of node importance are shown in Figure 13. In Figure 13a, the fault type is chipped tooth (cracks on the gear), and the model considers nodes 5 and 6, the vibration signals of the reduction gearbox shaft, to best reflect this fault condition. Figure 13b shows the fault type as normal operation (health working state). The model identifies nodes 0, 1, 2, and 3 as strongly indicative of this fault condition. Specifically, by analyzing the motor vibration signals and the x-, y-, and z-axis vibration signals of the planetary gearbox, it is possible to accurately determine whether the gear has failed. This aligns with previous studies that often used the vibration signals from columns 2, 3, and 4 to assess gear faults. Figure 13c shows the fault type as a missing tooth, similar to a gear with cracks. Node 5 has the highest feature contribution. However, in cases of damage, the model considers the abnormalities of nodes 5 and 6 to be the primary basis for determining the fault. When determining whether a gear has suffered a missing tooth fault, nodes 5 and 7 have higher contribution degrees, indicating that the model considers abnormalities in motor torque signals and planetary gearbox shaft vibration signals to be potential indicators of a missing tooth fault. Figure 13d indicates that the model primarily relies on the planetary gearbox shaft vibration signal to determine whether a root crack fault has occurred in the gear. Figure 13e indicates that when the motor torque signal and the reducer gearbox shaft vibration signal are abnormal, the model considers a surface wear fault to have occurred.

**Figure 14:**
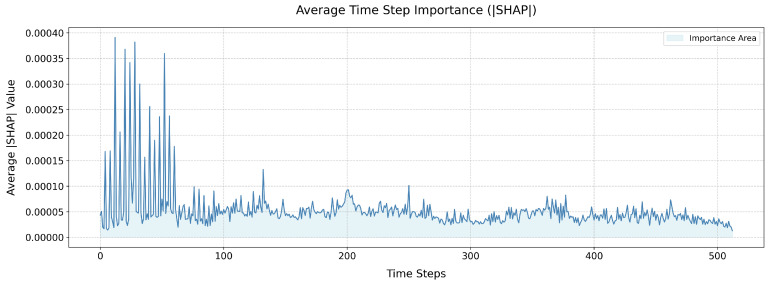
Average time step importance.

Figure 14 shows the average time step importance map of the diagnostic model. The information in the figure indicates that signal features at earlier time steps contribute more to model diagnosis, which shows that the model can effectively capture the time-dependent characteristics between data and can identify gear failures at earlier time steps (<100).

**Figure 15:**
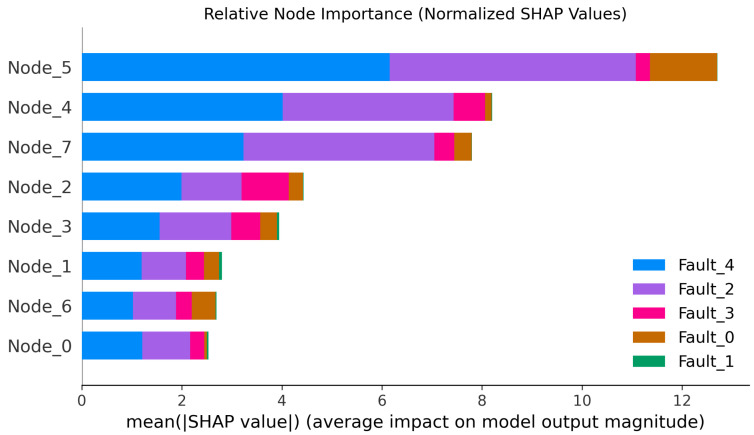
Summary bar.

Figure 15 shows the average importance of each node for the model diagnostic logic. For the PI-Dual-STGCN fault diagnosis model proposed in this paper. Nodes 5, 4, and 7 are considered the most important node features during model diagnosis, meaning that the model focuses more on whether the signals from the x-axis of the reduction gearbox, the motor torque signal, and the y-axis of the reduction gearbox are abnormal when determining the type of fault that has occurred. In the figure, Fault_1 represents the gear health category. Since all sensor detection signals are normal when the gear is operating normally, no single node exhibits an abnormally high response to the health category, resulting in Fault_1 having a relatively small proportion in the bar charts of each node in the figure.

**Figure 16:**
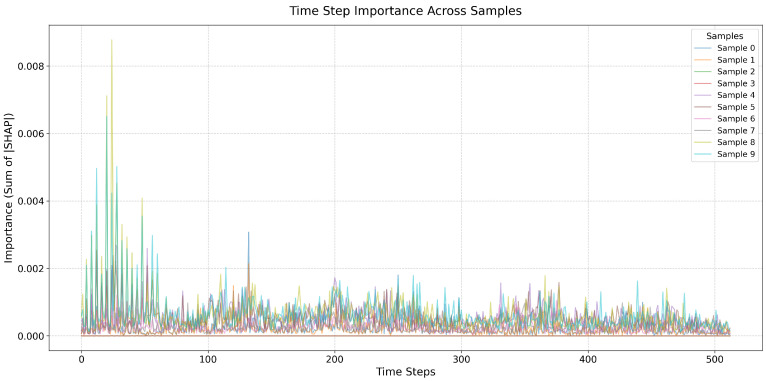
Consistency assessment of time step importance.

After performing interpretable analysis on the model using SHAP, the SHAP evaluation metrics designed in Section 2 were used to assess the accuracy of SHAP’s interpretability. The evaluation results are shown in Table 5. SHAP provides excellent interpretability for node and time step importance, with metrics approaching 1. As shown in Figure 16, the results of the time importance consistency assessment were obtained by using a hierarchical random sampling method to sample two samples for each of the five faults, totaling 10 samples. The consistency of the temporal step importance curves across different samples is high, indicating that the explanatory algorithm performs well.

**Figure 17:**
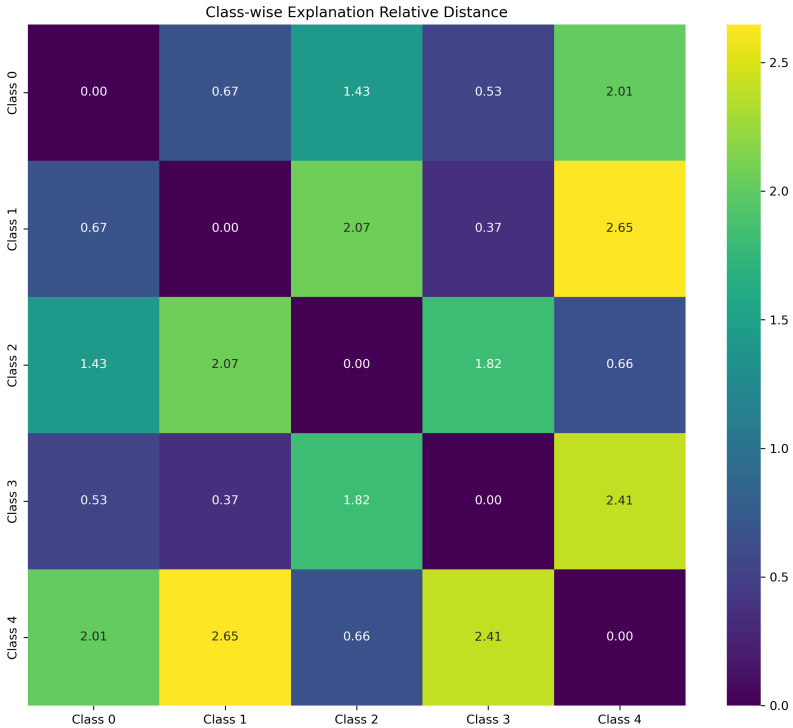
Visual representation of category distinctions by the algorithm.

The interpretability stability of the algorithm also tends toward 1, indicating that the model produces consistent results for the same fault sample even when the background data differs, ensuring the reproducibility of the interpretability algorithm. The rationality metric is 0.800, which is relatively low compared to the previous metrics but still indicates that the interpretation of the model is reasonably sound. The author believes that the relatively low value may be due to the fact that, when calculating the rationality metric, only the node data with the highest feature contribution is set to zero. However, as shown in Figure 13a,c,e, under fault conditions such as missing teeth, missing teeth, and tooth surface wear, node 5 accounts for the highest proportion of importance. Therefore, with the original metric design, the rationality assessment value is relatively low. The visualization effect of category distinction is shown in Figure 17, where different values correspond to different color intensities. There are obvious color differences between categories in the figure, indicating that the explanatory algorithm can effectively distinguish between different fault categories.

### 4.2. Model Diagnosis Text Explanation Based on RAG and LLM

**Figure 18:**
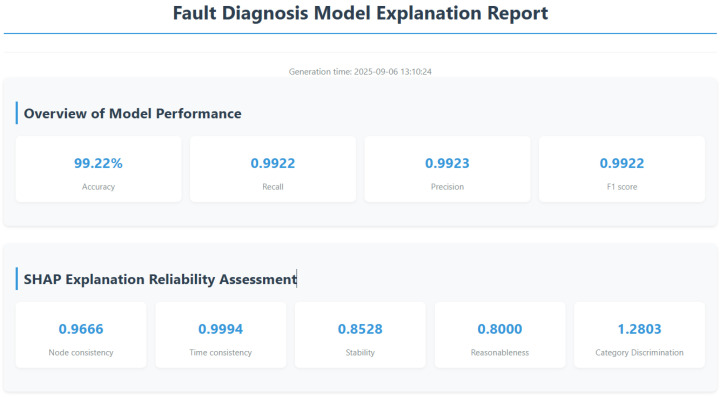
Visualization of key indicators in the main report.

**Figure 19:**
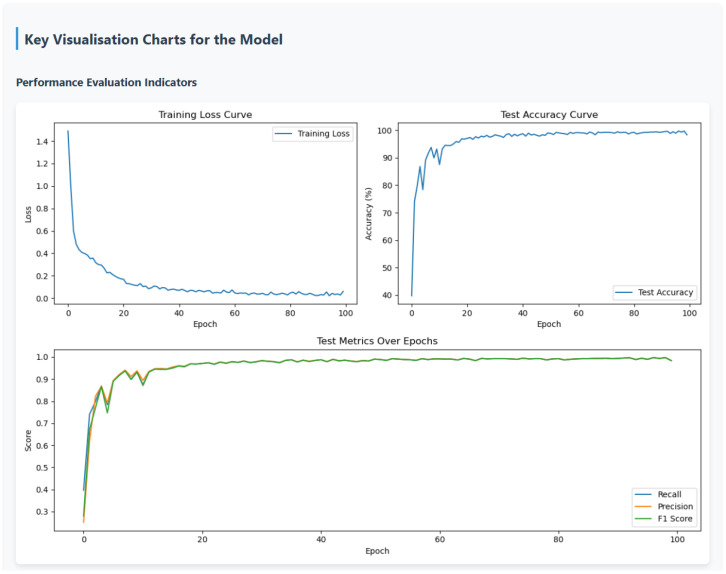
Key charts from the main report visualized.

**Figure 20:**
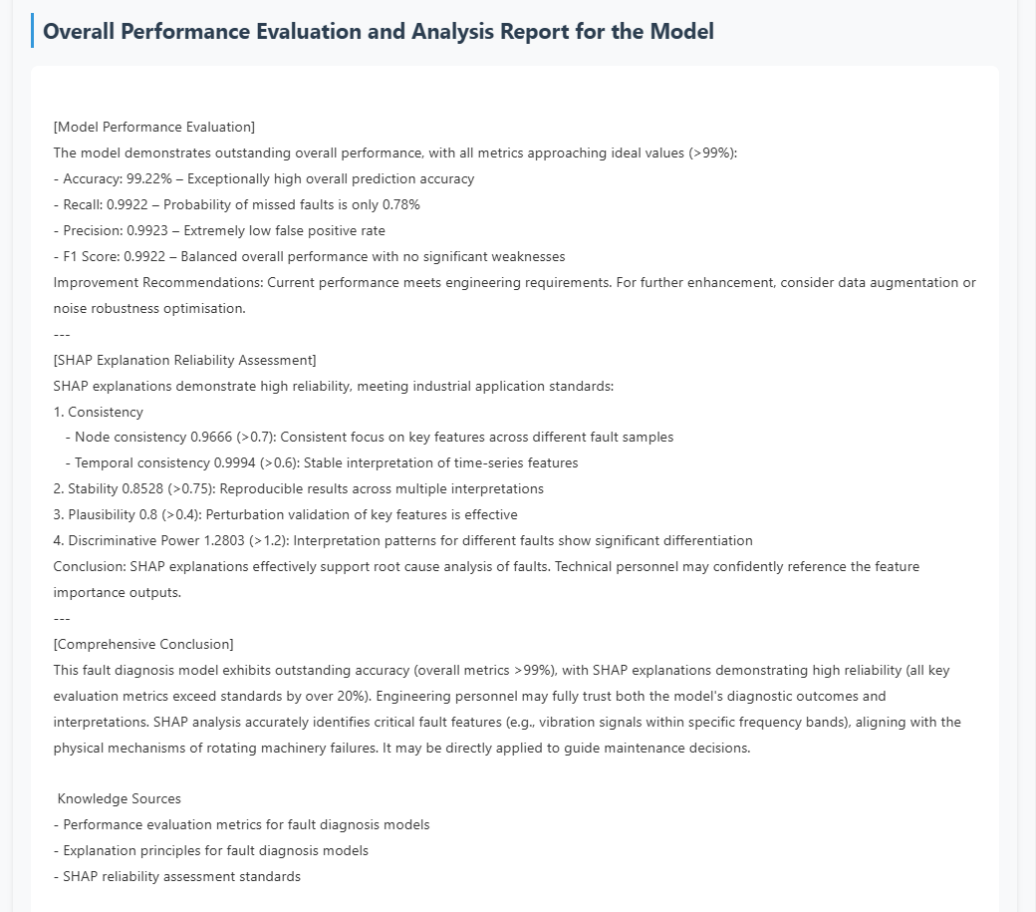
Main analysis report visualization.

**Figure 21:**
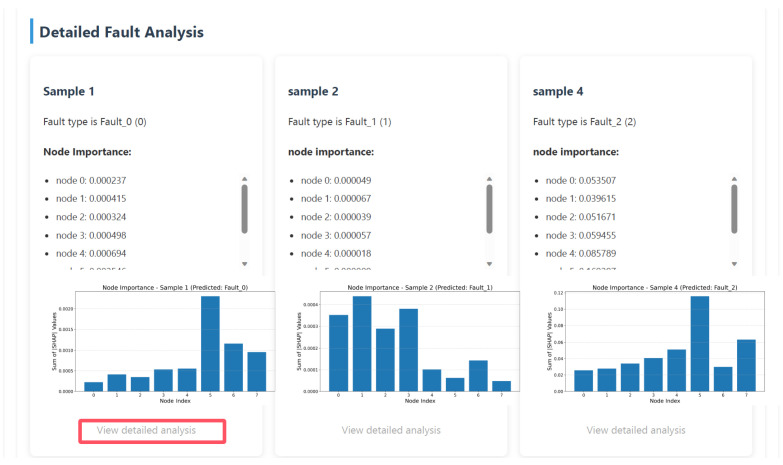
Jump link from main report to sub-report, with red boxes marking the link button for jumping to the sub-report.

The model diagnostic text explanation based on RAG and and the LLM includes a main report and a sub-report. The main report first highlights the model’s performance parameters and reliability assessment indicators of the explanation algorithm, as shown in Figure 18. It then visually presents the key charts of the model and the explanatory algorithm, as shown in Figure 19. Additionally, RAG technology is used to retrieve model performance evaluation standards and SHAP reliability assessment standards from the knowledge base. Combined with the powerful reasoning capabilities of the LLM, the model and explainable algorithms are evaluated and analyzed, as shown in Figure 20, significantly enhancing the confidence of engineering technicians in using this model and explainable algorithms. Meanwhile, the main report displays a summary of the sub-report and a jump link, as shown in Figure 21.

**Figure 22:**
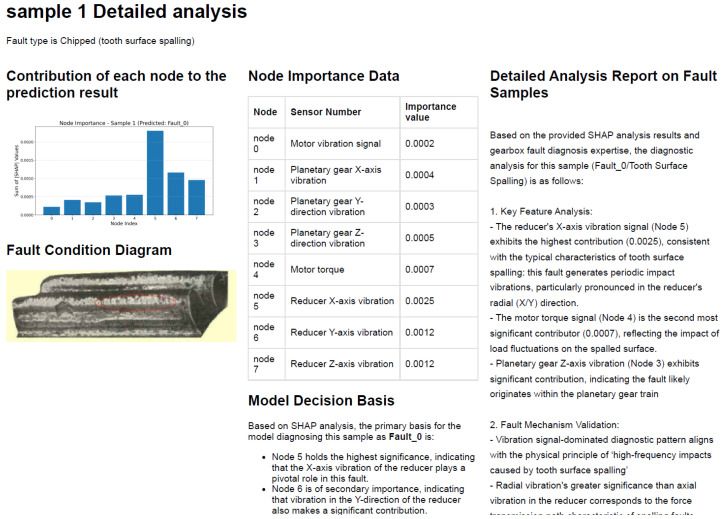
Sub-report results display (partial view), with red circles marking specific examples of gear damage.

As shown in Figure 22, in the sub-report, RAG technology is used to retrieve fault types and related experience knowledge from the knowledge base, providing key visualization charts, node importance data, fault type graphs, model decision-making basis, and detailed analysis reports on fault samples, key feature analysis, and maintenance recommendations provided by the LLM.

## 5. Conclusions

This paper proposes a new PI-Dual-STGCN fault diagnosis model and introduces a general SHAP-LLM joint explanation framework for explaining deep learning algorithms. The former incorporates physical topological structures into the graph construction process, effectively enhancing the physical interpretability of graph representations. Experimental results demonstrate that physical information graphs and signal association graphs are complementary, with their synergistic use achieving up to a 1.31% improvement in model performance. The latter is a general, highly scalable new model interpretability framework. Given the severe hallucinations and inherent opacity of current large models, this study did not adopt conventional methods using large models or multimodal large models to perform fault diagnosis tasks. Instead, it employed relatively mature deep learning algorithms for fault diagnosis tasks. XAI technology is used to explain the diagnostic behavior logic of the model, providing visual explanations, while corresponding evaluation metrics are designed for reliability assessment. Ultimately, an external knowledge base is constructed, encompassing model performance evaluation standards, explainability algorithm reliability evaluation standards, fault diagnosis, and related experience. The model’s performance metrics and SHAP reliability metrics are presented in the main report prompt, and RAG technology is combined to evaluate the reliability of the model’s performance and the explainability algorithm, with the results presented in the main report. The quantitative explanation information of SHAP on the diagnostic model’s behavioral logic is provided in the sub-report prompt. Combined with RAG technology, detailed analysis of fault samples is conducted, providing fault details and necessary maintenance decision recommendations. Through experimental verification, the PI-Dual-STGCN fault diagnosis model proposed in this paper can effectively complete fault diagnosis tasks. Additionally, the SHAP-LLM joint explanation framework has universality, providing an innovative explainable mechanism for the industrial fault diagnosis field.

It should be noted that the current study has several key limitations that restrict its comprehensive applicability and further exploration of the proposed framework:

- While the Table 3 has confirmed the effectiveness of the designed physical prior graph (model performance decreases notably when this component is removed), this study only adopted one specific construction strategy—based on the gearbox’s motion transmission paths and component co-location—for the physical prior graph. Alternative, potentially more optimal construction schemes (e.g., incorporating finer-grained physical constraints such as gear meshing stiffness) that could enhance the graph’s physical interpretability and the model’s diagnostic performance remain unexplored.
- Due to manuscript space constraints, validation of the framework was not extended to scenarios representative of practical industrial deployments, including sensor re-layout, partial missing sensors, or cross-platform sensor configurations. The robustness of the physical prior graph (and the overall PI-Dual-STGCN model) under these non-ideal sensing conditions is thus untested.
- Although the SEU gearbox dataset used in the experiment has five types of faults, the fault data is relatively independent and does not include fault samples where multiple faults occur simultaneously. Therefore, the performance of the PI-Dual-STGCN model and the SHAP-LLM framework in the compound fault scenario (where multiple defects exist in the gearbox) has not yet been verified. This limits the direct application value of the proposed method in real industrial scenarios—where the phenomenon of multi-defect degradation is widespread.
- The current SHAP-LLM joint interpretation only focuses on data-level feature importance, and a correspondence table linking fault mechanisms (e.g., gear meshing anomalies), observable physical features (e.g., meshing frequencies, sidebands, envelope features), and SHAP high-importance regions has not been established. Additionally, we did not conduct cross-validation with alternative XAI methods (e.g., LIME), which restricts verification of the interpretability results’ physical rationality, consistency, and stability.

In future research, we will first address the identified limitations by exploring diverse construction strategies for the physical prior graph—incorporating finer-grained physical constraints such as gear meshing stiffness and adaptive initialization schemes—and systematically evaluating their impacts on the graph’s physical interpretability and the PI-Dual-STGCN model’s diagnostic performance. Subsequently, we will simulate or deploy experimental setups involving sensor re-layout, partial missing sensors, and cross-platform configurations to validate the robustness of the physical prior graph and the overall model under non-ideal industrial sensing conditions while expanding the experimental dataset to include composite fault samples for verifying the framework’s performance in multi-defect scenarios. We will explore methods for integrating physical knowledge with loss functions to further enhance the inherent interpretability of the PI-Dual-STGCN model and incorporate envelope spectral analysis information into the SHAP-LLM joint explanation framework to verify model performance and interpretation reliability from multiple angles, ultimately providing more detailed and scientific fault explanations and maintenance recommendations. Beyond these steps, we will: (1) establish a correspondence table linking fault mechanisms, observable physical features (e.g., meshing frequencies, sidebands), and SHAP high-importance regions, to enhance the physical rationality of interpretation; (2) introduce at least one alternative XAI method for cross-validation, to assess the consistency and stability of interpretability results; (3) explore physical knowledge-integrated loss functions to enhance the PI-Dual-STGCN model’s inherent interpretability and incorporate envelope spectral analysis into the SHAP-LLM framework. Subsequent research will focus on the physical plausibility of its interpretability, adaptability to engineering scenarios, and the integration mechanism between large language models and external engineering knowledge (enabling non-specialists to quickly understand and trust algorithmic results). The ultimate goal is to provide more detailed, scientifically grounded, and engineering-friendly fault explanations and maintenance recommendations.

## Figures and Tables

**Table 1:** Hyperparameters and experimental settings for PI-Dual-STGCN.

Category	Parameter/Component	Value/Specification
Data Preprocessing	Window size	1024 sampling points
	Sliding step size	1024 sampling points (no overlap between samples)
	FFT output dimension	513 frequency-domain points
	Data split	80% training/20% testing (stratified random sampling)
	Normalization method	Min–Max normalization
Hyperparameters	Difference layers	4
	Node features	5
	Number of classes	5
	Number of nodes	8
	Learning rate	$1\times 10^{-4}$
	Weight decay	$1\times 10^{-5}$
	Batch size	64
	Number of epochs	80
	Optimizer	Adam
	Loss function	Cross-entropy
	Background dataset size	180
	Number of explanation samples	10
	Background sampling strategy	Hierarchical (Algorithm 1)
Hardware Specifications	CPU	Intel i7-9750H (2.6 GHz, 6 cores)
	GPU	NVIDIA GeForce RTX 2070 (8 GB VRAM)
	RAM	16 GB DDR4
Software Environment	Operating system	Ubuntu 20.04 LTS
	Python version	3.8.10
	PyTorch version	1.9.0
	CUDA version	11.4

**Table 2:** Performance comparison of different models in fault diagnosis tasks using the SEU dataset.

Models	Acc (Mean ± SD,%)	P (Mean ± SD,%)	R (Mean ± SD,%)	F1 (Mean ± SD,%)
MCN-LSTM	95.87 ± 0.62	96.10 ± 0.58	94.85 ± 0.67	95.47 ± 0.60
GAT	96.22 ± 0.51	97.85 ± 0.47	97.15 ± 0.53	97.50 ± 0.49
GGCN	98.15 ± 0.38	98.30 ± 0.35	97.85 ± 0.41	98.07 ± 0.37
HAGCN	98.34 ± 0.32	98.45 ± 0.29	98.10 ± 0.34	98.27 ± 0.30
AM-GCN	99.05 ± 0.18	99.12 ± 0.16	98.95 ± 0.20	99.03 ± 0.17
MNQGN	97.82 ± 0.43	98.05 ± 0.40	97.25 ± 0.46	97.64 ± 0.42
MRF-GCN	98.76 ± 0.27	98.85 ± 0.25	98.60 ± 0.29	98.75 ± 0.26
PI-Dual-STGCN (Ours)	99.22 ± 0.09	99.22 ± 0.08	99.2 ± 0.10	99.22 ± 0.08

**Table 3:** Ablation experiment study.

Variant Models	Acc (Mean ± SD,%)	P (Mean ± SD,%)	R(Mean ± SD,%)	F1 (Mean ± SD,%)	ΔF1
Full Model (Ours)	99.22 ± 0.09	99.22 ± 0.08	99.20 ± 0.10	99.22 ± 0.08	-
w/o PI Graph	98.65 ± 0.21	98.70 ± 0.20	98.52 ± 0.22	98.61 ± 0.21	−0.61
w/o Signal Graph	98.71 ± 0.19	98.75 ± 0.18	98.63 ± 0.20	98.68 ± 0.19	−0.54
Single-Graph	97.95 ± 0.33	98.00 ± 0.31	97.82 ± 0.35	97.91 ± 0.33	−1.31
w/o MS-Diff	98.88 ± 0.15	98.90 ± 0.14	98.82 ± 0.16	98.86 ± 0.15	−0.36

**Table 4:** Quantitative metrics for over-smoothing and representation quality.

Variant Models	Average Inter-Layer Node Embedding Similarity	Intra-/Inter-Class Distance Ratio	SHAP Score Stability (Standard Deviation)
Full Model (Ours)	0.63	0.47	0.08
w/o MS-Diff	0.71	0.51	0.10

Note: (1) Higher inter-layer node embedding similarity indicates more severe feature homogenization (over-smoothing); (2) lower intra-/inter-class distance ratio reflects better separability of fault class representations; (3) smaller SHAP score standard deviation corresponds to more stable model decision explanation.

**Table 5:** Reliability performance of the SHAP algorithm explanation.

Evaluation Indicator Name	Indicator Value
Node importance consistency	0.9666
Time step importance consistency	0.9994
Interpretation stability	0.9996
Interpretation rationality	0.8008
Category discrimination	1.4624

## Data Availability

The datasets used in this study are publicly available on GitHub and official websites. The dataset is available at https://github.com/cathysiyu/Mechanical-datasets (Commit ID: 9732a28, accessed on 15 June 2025).
